# NO Oxidation States in Nonheme Iron Nitrosyls: A DMRG-CASSCF Study of {FeNO}^6–10^ Complexes

**DOI:** 10.1021/acs.inorgchem.4c03845

**Published:** 2025-01-23

**Authors:** Quan Manh Phung, Ho Ngoc Nam, Vic Austen, Takeshi Yanai, Abhik Ghosh

**Affiliations:** † Department of Chemistry, Graduate School of Science, Nagoya University, Furo-cho, Chikusa-ku, Nagoya, Aichi 464-8602, Japan; ‡ Institute of Transformative Bio-Molecules (WPI-ITbM), Nagoya University, Furo-cho, Chikusa-ku, Nagoya, Aichi 464-8601, Japan; § Department of Materials Process Engineering, Graduate School of Engineering, Nagoya University, Furo-cho, Chikusa-ku, Nagoya 464-8603, Japan; ∥ Department of Chemistry, UiT The Arctic University of Norway, N-9037 Tromso̷, Norway

## Abstract

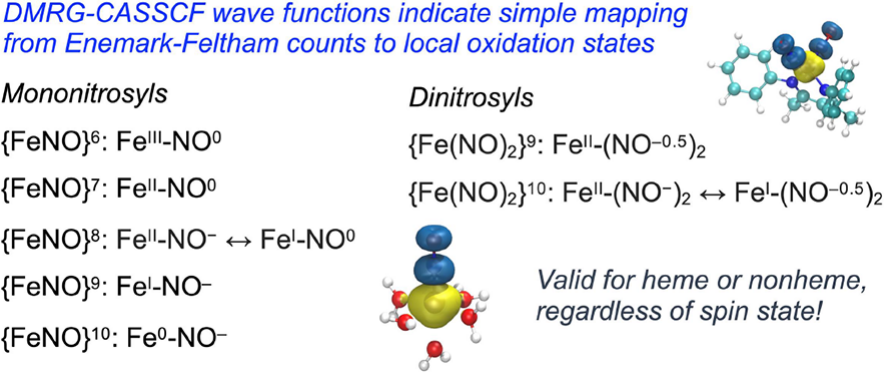

Building upon an earlier study of heme-nitrosyl complexes (*Inorg. Chem*. **2023**, *62*, 20496–20505), we examined a wide range of nonheme {FeNO}^6–10^ complexes (the superscript represents the Enemark-Feltham count) and two dinitrosyl iron complexes using DMRG-CASSCF calculations. Analysis of the wave functions in terms of resonance forms with different [π*(NO)]^*i*^ occupancies (where *i* = 0–4 for mononitrosyl complexes) identified the dominant electronic configurations of {FeNO}^6^ and {FeNO}^7^ complexes as Fe^III^–NO^0^ and Fe^II^–NO^0^, respectively, mirroring our previous findings on heme-nitrosyl complexes. A trigonal-bipyramidal *S* = 1 {FeNO}^8^ complex with an equatorial triscarbene ligand set appears best described as a resonance hybrid of Fe^I^–NO^0^ and Fe^II^–NO^–^. Reduction to the corresponding *S* = 1/2 {FeNO}^9^ state was found to involve both the metal and the NO, leading to an essentially Fe^I^–NO^–^ complex. Further reduction to the {FeNO}^10^ state was found to be primarily metal-centered, leading to a predominantly Fe^0^–NO^–^ configuration. Based on the weights *w*_*i*_ of the [π*(NO)]^*i*^ resonance forms, an overall DMRG-CASSCF-based π*(NO) occupation number could be derived, which was found to exhibit a linear correlation with both the NO bond distance and NO stretching frequency, allowing a readout of the NO oxidation state from the NO bond distance.

## Abstract 2

Analysis of the DMRG-CASSCF wave functions for a wide range of iron nitrosyls has allowed the determination of long-sought local oxidation states of the Fe center and the NO moiety. The picture that emerges appears to hold for heme and nonheme systems alike and regardless of spin state for a given Enemark-Feltham count.

## Introduction^1^

Nitric oxide has long been the exemplar of a so-called noninnocent ligand,^2,3^ i.e., a ligand that does not allow one to readily determine the oxidation state of a metal center to which it is bound.^4,5^ The reason lies in the highly covalent nature of the metal(d)-NO(π*) interaction, which hinders the application of the ionic approximation to the metal-NO linkage.^6−10^ The popular Enemark-Feltham formalism elegantly sidesteps the question of metal and NO oxidation states by describing a metal-nitrosyl unit with an effective d electron count *n* defined as the number of metal d electrons plus the number of NO π* electrons.^11^ The question of local oxidation states, especially the oxidation state of a bound NO ligand, however, *is* an important one: it lies, for instance, at the heart of the question whether a given redox process is centered on the metal, the NO, or both. Recently, we reported a potential solution to the problem for heme (i.e., iron porphyrin) nitrosyl complexes with *n* = 6–8 by analyzing their DMRG-CASSCF wave functions in terms of localized orbitals.^12^ Our analysis indicated (a) NO^0^ resonance forms [i.e., Fe(II)–NO^0^ and Fe(III)–NO^0^] as the major contributors to both {FeNO}^6^ and {FeNO}^7^ complexes and (b) an Fe(I)–NO^0^ ↔ Fe(II)–NO^–^ resonance hybrid description for {FeNO}^8^ anion ^1^{Fe[P](NO)}^−^ (where the superscripted numeral prefix refers to the spin multiplicity and P to the unsubstituted porphyrin ligand). Herein, we have attempted to extend our analysis to nonheme iron nitrosyls (in which the iron center is not encapsulated by a porphyrin-type ligand).

**Chart 1:**
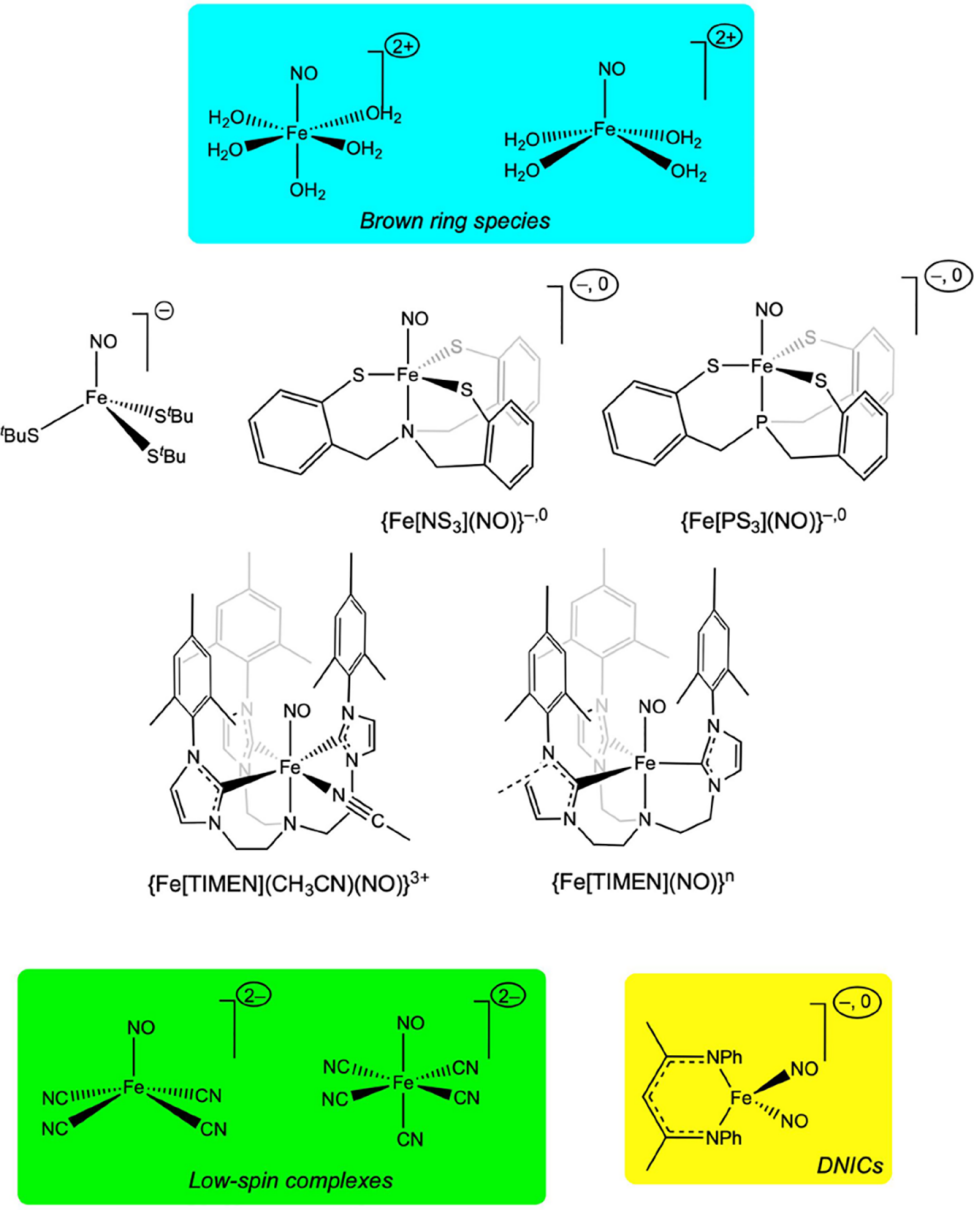
Nonheme-Nitrosyl Complexes Investigated in This Work

Freed of the geometric constraints imposed by a porphyrin, mononuclear iron nitrosyls (MNICs) can exhibit a much wider variety of coordination geometries as well as oxidation and spin states.^2,3^ Thus, nonheme {FeNO}^6,7^ complexes may be either low- or high-spin, unlike their heme counterparts, which are invariably low-spin. Here, accordingly, we have examined several *S* = 3/2 {FeNO}^7^ complexes including two models of the famous “brown-ring complex”, the classic ^4^{Fe(H_2_O)_5_(NO)}^2+^ complex^13^ and a newly proposed ^4^{Fe(H_2_O)_4_(NO)}^2+^ form (which has been suggested to coexist with the classic form^14^), and three Fe-thiolate-NO complexes, ^4^{Fe(S^*t*^Bu)_3_(NO)}^−^,^15^^4^{Fe[NS3](NO)}^−^ and ^4^{Fe[PS3](NO)}^−^ (Chart 1).^16^ For the latter three species, we also examined the oxidized *S* = 1 {FeNO}^6^ states. In addition, we have examined the low-spin complexes ^2^[Fe(CN)_4_(NO)]^2–^^17,18^ and ^1^[Fe(CN)_5_(NO)]^2–^ (i.e., nitroprusside).^19−21^ Of these, ^2^[Fe(CN)_4_(NO)]^2–^ provides an unusual example of a low-spin square-pyramidal {FeNO}^7^ complex with a strictly linear NO, which may be contrasted with FeNO angles ∼140° in low-spin nitrosylhemes. The unusual linearity of low-spin {FeNO}^7^ units in certain square-pyramidal environments is thought to reflect Fe(3d_*z*2_-4p_*z*_) mixing facilitated by a pyramidalized FeNO geometry (note: additional examples have emerged in recent years^22^). In re-examining these systems with DMRG-CASSCF calculations, we have sought to determine to what extent our earlier findings on heme-nitrosyl systems are transferable to the far more diverse world of nonheme iron nitrosyls.

A second major goal was to better understand the nature of the highly reduced {FeNO}^8–10^ states, which have recently been accessed with tripodal triscarbene^23^ and trisphosphine^24^ ligands and which are unprecedented in heme systems. Here we have chosen to apply our methodology to the *t*ris[2-(3-mesityl*im*idazol-2-ylidene)*e*thyl]ami*n*e- (TIMEN) based {FeNO}^6–10^ series reported by Meyer and co-workers (Chart 1).^23^

Third and finally, we have re-examined the nature of the dinitrosyl iron complexes (DNICs) ^2^{Fe[nacnac](NO)_2_} and ^1^{Fe[nacnac](NO)_2_}^−^ (Chart 1), which also exemplify high Enemark-Feltham counts, {Fe(NO)_2_}^9^ and {Fe(NO)_2_}^10^, respectively. Initially reported by Lippard and co-workers,^25^ these and analogous complexes have subsequently been the subject of multiple detailed electronic-structural studies.^26,27^

## Methods

As in our earlier work on heme-nitrosyl complexes, all structures were optimized at the BP86-D3(BJ)/def2-TZVP level of theory using the Turbomole v.7.4 software package.^28−33^ For {Fe(H_2_O)_5_(NO)}^2+^, we found that the key structural parameters, such as bond lengths and angles in the Fe–NO unit, are not very sensitive with respect to the employed functional (Table S7). We observed that the variations in functional choice led to minimal changes in optimized bond lengths (within ∼0.04 Å) and bond angles (within one degree). Single-point state-specific DMRG-CASSCF calculations^34−44^ were then performed on the BP86 optimized geometries using the same basis set (def2-TZVP) and the interface between CheMPS2 1.8.12 and OpenMolcas v11.9 interface.^45−48^ Given that our analysis does not depend on an explicit treatment of dynamic correlation, the use of larger basis sets was found to be unnecessary. Two-electron integrals were computed using the Cholesky decomposition technique with a threshold of 10^–6^ au.^49^ The number of renormalized states *m* was set to 1000, which should be sufficient to capture the multiconfigurational character effectively without significant truncation error.^50^ For the largest active space (25 active orbitals), a test calculation with *m* = 1500 validates this observation (Table S64).

The active spaces for each complex are summarized in Table 1. The active spaces consist of all Fe(3d) orbitals, a set of Fe(4d) orbitals to describe the double-shell effect (see Table S1),^51,52^ all Fe-ligand σ orbitals (if applicable), and a set of ten NO-based orbitals. The latter set comprises two π(NO) orbitals and the corresponding π*(NO) orbitals, the σ(NO) orbital and the corresponding σ*(NO) correlating orbital, and two π′(NO) orbitals to capture the radial correlation of the π*(NO) orbitals. Table 1 summarizes the formal electronic configurations of the complexes studied, while visual representations of the natural active orbitals are provided in Figures S1–S17.

**Table 1:** Formal Electron Configuration of Complexes Studied in This Work

electron configuration^a^	complex	active space^b^
{FeNO}^6^		
[d_*xz*_ + π*(NO)]^2^ [d_*yz*_ + π*(NO)]^2^ (d_*xy*_)^↑^ (d_*x*2*–y*2_)^0^ (d_*z*2_)^↑^	^3^{Fe(S^*t*^Bu)_3_(NO)}	14 in 18
		
[d_*xz*_ + π*(NO)]^2^ [d_*yz*_ + π*(NO)]^2^ (d_*xy*_)^↑^ (d_*x*2*–y*2_)^↑^ (d_*z*2_)^0^	^3^{Fe[NS3](NO)}	18 in 20
	^3^{Fe[PS3](NO)}	
		
[d_*xz*_ + π*(NO)]^2^ [d_*yz*_ + π*(NO)]^2^ (d_*xy*_)^2^ (d_*x*2*–y*2_)^0^ (d_*z*2_)^0^	^1^{Fe(CN)_5_(NO)}^2–^	16 in 18
	^1^{Fe[TIMEN](CH_3_CN)(NO)}^3+^	
		
{FeNO}^7^		
[d_*xz*_ + π*(NO)]^2^ [d_*yz*_ + π*(NO)]^2^ (d_*xy*_)^↑^ (d_*x*2*–y*2_)^↑^ (d_*z*2_)^↑^	^4^{Fe(H_2_O)_5_(NO)}^2+^	13 in 18
	^4^{Fe(H_2_O)_4_(NO)}^2+^	
	^4^{Fe(S^*t*^Bu)_3_(NO)}^−^	
	^4^{Fe[NS3](NO)}^−^	
	^4^{Fe[TIMEN](NO)}^2+^	
		
[d_*xz*_ + π*(NO)]^2^ [d_*yz*_ + π*(NO)]^2^ (d_*xy*_)^2^ (d_*x*2*–y*2_)^↑^ (d_*z*2_)^0^	^2^{Fe[PS3](NO)}^−^	13 in 18
		
[d_*xz*_ + π*(NO)]^2^ [d_*yz*_ + π*(NO)]^2^ (d_*xy*_)^2^ (d_*x*2*–y*2_)^0^ (d_*z*2_)^↑^	^2^{Fe(CN)_4_(NO)}^2–^	15 in 18
		
{Fe(NO)_*n*_}^8–10^		
[d_*xz*_ + π*(NO)]^2^ [d_*yz*_ + π*(NO)]^2^ (d_*xy*_)^2^ (d_*x*2*–y*2_)^↑^ (d_*z*2_)^↑^	^3^{Fe[TIMEN](NO)}^+^	14 in 18
[d_*xz*_ + π*(NO)]^2^ [d_*yz*_ + π*(NO)]^2^ (d_*xy*_)^2^ (d_*x*2*–y*2_)^2^ (d_*z*2_)^↑^	^2^{Fe[TIMEN](NO)}	15 in 18
[d_*xz*_ + π*(NO)]^2^ [d_*yz*_ + π*(NO)]^2^ (d_*xy*_)^2^ (d_*x*2*–y*2_)^2^ (d*z*_2_)^2^	^1^{Fe[TIMEN](NO)}^−^	16 in 18
[d_*xz*_ + π*(NO) + π*(NO)]^2^ [d_*yz*_ + π*(NO) + π*(NO)]^2^ (d_*xy*_)^2^ (d_*x*2*–y*2_)^↑^ (d_*z*2_)^2^	^2^{Fe[nacnac](NO)_2_}	21 in 25
[d_*xz*_ + π*(NO) + π*(NO)]^2^ [d_*yz*_ + π*(NO) + π*(NO)]^2^ (d_*xy*_)^2^ (d_*x*2*–y*2_)^2^ (d_*z*2_)^2^	^1^{Fe[nacnac](NO)_2_}^−^	22 in 25

a For nonaxial FeNO complexes, the distinction between d_*xz*_ and d_*z*2_ orbitals is unclear.

b The notation “*n*_*e*_ in *n*_*a*_*”* was used to denote an active space of *n*_*e*_ electrons in *n*_*a*_ orbitals.

The DMRG-CASSCF wave functions were analyzed in terms of localized orbitals.^12,53−56^ First, all DMRG-CASSCF natural orbitals were localized to NO-based and Fe-based orbitals. Specifically, after obtaining DMRG-CASSCF natural orbitals, we applied the Pipek–Mezey localization method to bonding and antibonding orbitals between Fe 3d and NO 2p orbitals, as well as Fe 4d and O 3p orbitals, resulting in a clear distinction between Fe- and NO-based localized orbitals. The remaining DMRG active orbitals were already fairly localized and did not require further adjustment. Subsequently, we employed the BLOCK2 code to decompose the wave function into configuration state functions (CSFs).^57,58^ Specifically, for each complex, we generated one- and two-electron integrals in the molecular orbital basis (FCIDUMP) with OpenMolcas and performed DMRG calculations with BLOCK2. For small active spaces of 18 active orbitals, the results from BLOCK2 (localized orbitals) and CheMPS2 (natural orbitals) agree closely (to within 10^–4^ Ha). In our mapping, we recovered at least 95% of the total CSF weight (for the large active space of 25 active orbitals) and up to 99% for the small active space of 18 active orbitals. The CSFs were then categorized into four resonance structures (Fe–NO^+^, Fe–NO^0^, Fe–NO^–^, and Fe–NO^2–^), enabling the determination of the local oxidation states for both Fe and NO. To examine the impact of variations in the localization process, we repeated the DMRG calculations using two extra iterative localization procedures (Boys and Edmiston–Ruedenberg localizations) for {Fe(H_2_O)_5_(NO)}^2+^. We found that the primary results—such as the percentage contributions of major resonance forms—were robust with respect to these variations (Table S6). Specifically, the largest weights were found to change less than ∼5%, indicating the stability of our findings with respect to the localization scheme. Similarly, the DMRG results proved relatively insensitive to minor structural variations arising from the use of different DFT optimizations (Table S8). Additionally, we calculated the DMRG-CASSCF spin densities and Mulliken spin populations. Since the CheMPS2-OpenMolcas interface does not provide Mulliken population analyses, atomic spin populations were calculated with the BLOCK code interfaced in the *orz* program package instead.^43^

## Results and Discussion

### Spin Density Profiles

**Figure 1:**
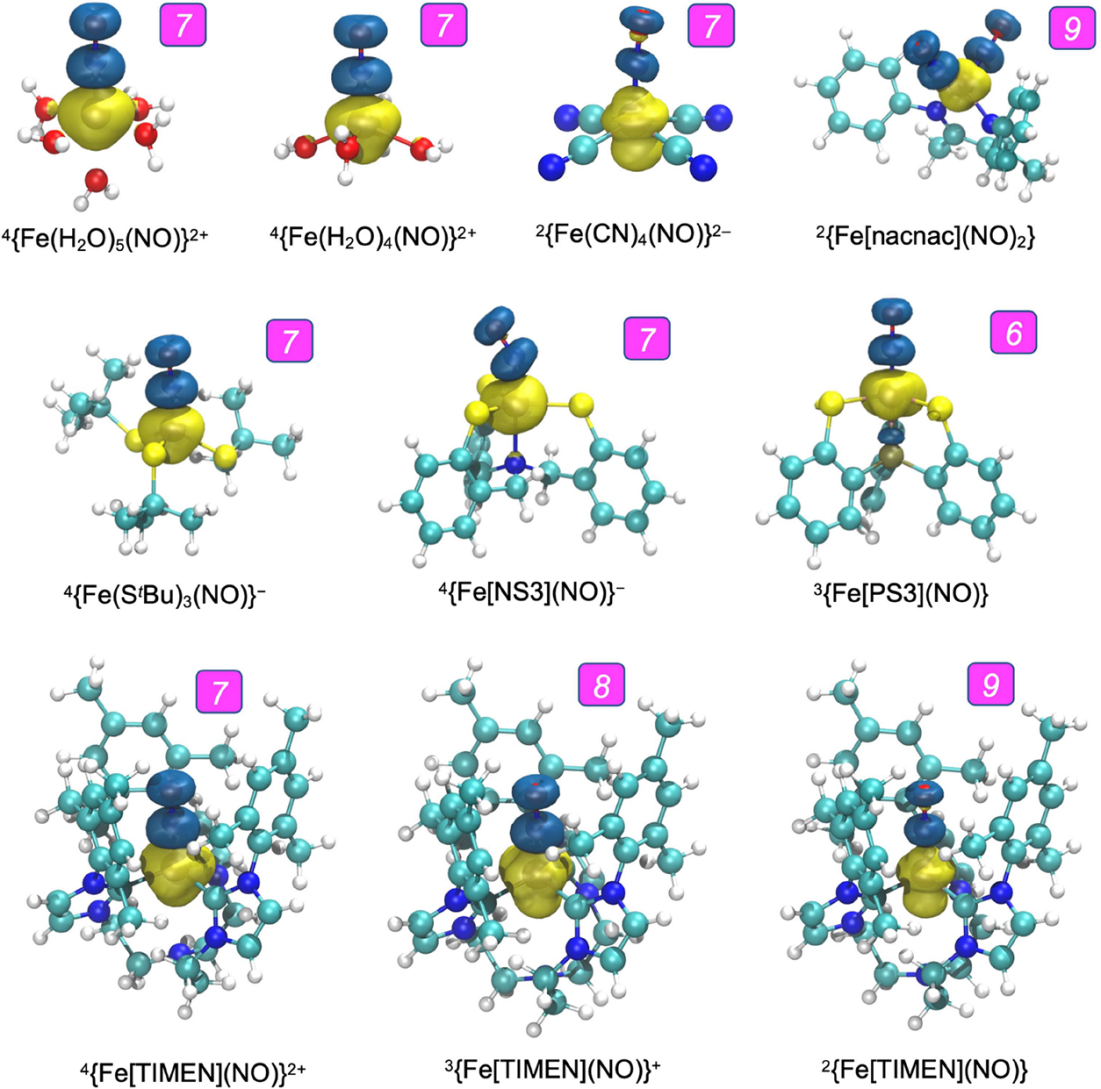
DMRG-CASSCF spin density plots of selected open-shell complexes. Enemark-Feltham counts are indicated by white numerals within magenta boxes.

Figure 1 presents DMRG-CASSCF spin densities for selected *S* > 0 ground-state species. We also calculated spin densities with several DFT methods using BP86-D3 optimized geometries. Mulliken spin populations for Fe, N, and O are listed in the Supporting Information for both DMRG-CASSCF and DFT methods. All the spin densities in Figure 1 (as well as those reported earlier for heme-nitrosyl systems^12^) appear to share two common features–a significant spatial separation of majority (say α) and minority (say β) spin densities and a dumbbell-like shape of the minority spin density localized around the NO vector. These two qualitative features are also shared by the DFT spin densities. Quantitatively, however, none of the exchange-correlation functionals reproduce the DMRG-CASSCF spin densities with great accuracy (due to various degrees of spin contamination), as may be judged from discrepancies in the Mulliken spin populations (see Supporting Information). Nevertheless, pure functionals such as BP86 appear to yield significantly better agreement with DMRG-CASSCF spin populations than hybrid functionals such as B3LYP.^59^ Another interesting point concerns the topology of the minority spin density: while the dumbbell-like shape may be suggestive of a locally triplet NO^–^ ligand, we shall see that such a formulation is simplistic, even though several of the complexes studied exhibit a degree of NO^–^ character. A significant dumbbell-like spin density on the NO moieties is also not expected for locally closed-shell NO^+^ ligands; as it happens, such an impression is indeed confirmed by the detailed analysis presented below.

### Analysis of the DMRG-CASSCF Wave Functions

**Figure 2:**
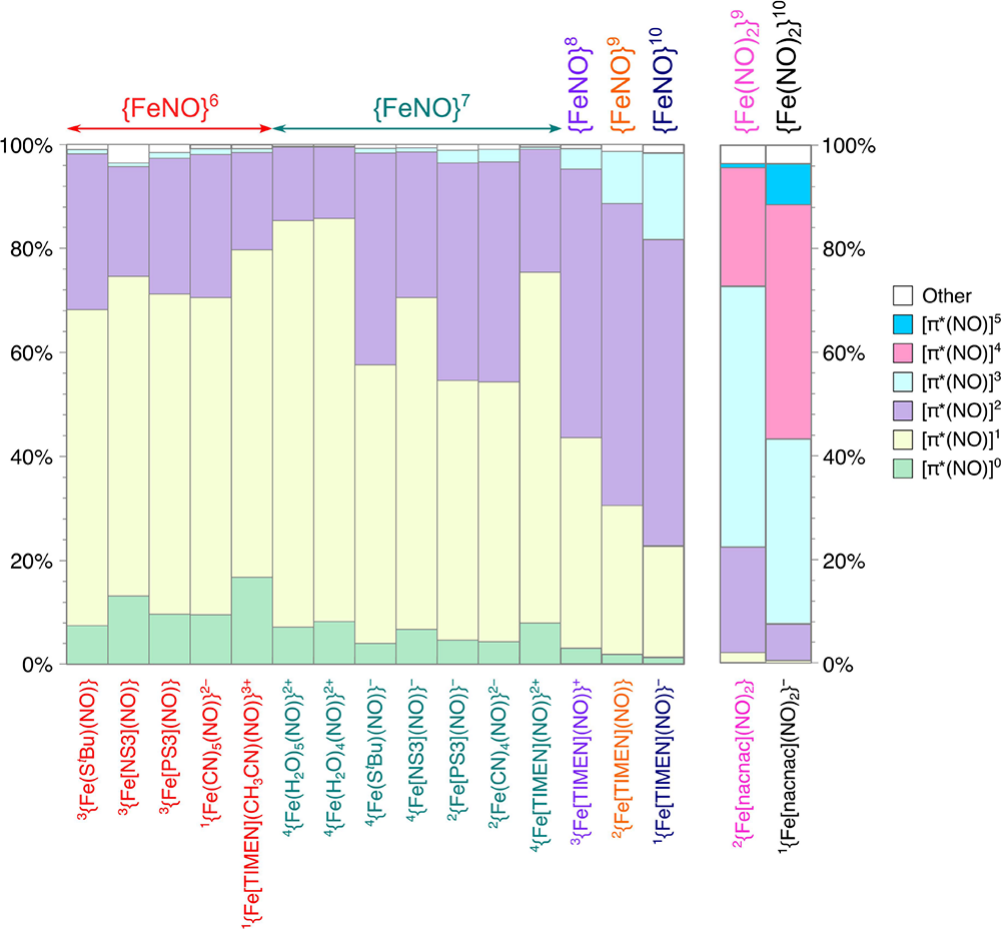
Weights (in percentage) of dominant configurations based on [π*(NO)]^*i*^ (*i* = 0, 1, 2, 3, ···) occupancy in DMRG-CASSCF wave functions.

To gain deeper insight into the electronic structure and bonding nature of the complexes, we analyzed the DMRG-CASSCF wave functions in terms of localized orbitals. This analysis decomposes the wave functions into configuration state functions (CSFs) that can be described as [π*(NO)]^*i*^ resonance forms, where *i*, the total number of electrons occupying the π*(NO) orbitals, can range from 0 to 4 for mononitrosyl complexes. An occupancy of 0 corresponds to NO^+^, while the complete filling of the π*(NO) orbitals (*i* = 4) signifies NO^3–^. It is worth emphasizing that a notation such as NO^0^ does not indicate a lone unpaired electron in a given π*(NO) orbital; rather, it signifies a combined electron density adding up to one electron distributed approximately equally between the two π*(NO) orbitals. For the dinitrosyl complexes, the combined occupation of the four π*(NO) orbitals can reach up to 8. The results of this analysis are presented in Figure 2, according to which the most significant configurations in nonheme-nitrosyl complexes, accounting for over 95% of the total wave functions, are [π*(NO)]^1^, [π*(NO)]^2^, and [π*(NO)]^0^.

#### {FeNO}^6^

A [π*(NO)]^1^ configuration appears to dominate for all the {FeNO}^6^ complexes studied, comprising more than 60% of the wave functions. The contribution of [π*(NO)]^2^ is smaller, being around 20–30%, followed by [π*(NO)]^0^ (around 5–15% of the wave functions). The contributions from [π*(NO)]^3^ and [π*(NO)]^4^ are negligible, being less than 1%. Interestingly, there seems to be no significant difference in NO resonance form composition between *S* = 0 nitroprusside and the*S* = 1 complex ^3^{Fe[PS3](NO)}. These results also mirror those observed for the {FeNO}^6^ heme complexes ^1^{Fe[P](NO)}^+^ and ^1^{Fe[P](NO)(ImH)}^+^ examined in our earlier study.^12^ Our DMRG-CASSCF results suggest that, all these complexes, whether *S* = 0 or *S* = 1, are best described as Fe^III^–NO^0^, which may be viewed as a departure from the description favored in earlier studies.^60^

#### {FeNO}^7^

In contrast to the relative uniformity of the {FeNO}^6^ systems, Figure 2 reveals substantial variations in configurational composition among the {FeNO}^7^ complexes examined. Although [π*(NO)]^1^ is the leading configuration for all species, its contribution varies over a large range, from a high of ∼78% in the celebrated brown-ring complex ^4^{Fe(H_2_O)_5_(NO)}^2+^, as well as in the corresponding five-coordinate species ^4^{Fe(H_2_O)_4_(NO)}^2+^ (which has been suggested to coexist in solution with the six-coordinate species), down to ∼50% in ^2^{Fe(CN)_4_(NO)}^2–^. With multiple strong-field ligands such as cyanide or thiolate, the [π*(NO)]^2^ configuration gains in importance, accounting for ∼40% of the total wave function for ^2^{Fe(CN)_4_(NO)}^2–^ and ^4^{Fe(S^*t*^Bu)(NO)}^−^; these complexes, as well as ^4^{Fe(NS3)(NO)}^−^, are thus best described as a resonance hybrid between Fe^II^–NO^0^ and Fe^III^–NO^–^ according to DMRG-CASSCF calculations. In contrast, the aqua complexes ^4^{Fe(H_2_O)_4,5_(NO)}^2+^, to a first approximation, can be described as Fe^II^–NO^0^.

#### TIMEN Series

Spanning five oxidation states from {FeNO}^6^ to {FeNO}^10^, the TIMEN series affords unusual insight into the question of metal versus NO-centered reduction.^23^ As for the other {FeNO}^6^ complexes examined here, an Fe^III^–NO^0^ description applies well for ^1^{Fe(TIMEN)(CH_3_CN)(NO)}^3+^. The {FeNO}^7^ complex appears best described as Fe^II^–NO^0^, corresponding to a largely metal-based reduction relative to the {FeNO}^6^ state. For the {FeNO}^8^ complex ^3^{Fe(TIMEN)(NO)}^+^, the [π*(NO)]^2^ configuration makes the most important contribution to the wave function (51.7%), with the [π*(NO)]^1^ configuration following at second place (40.5%). In other words, the {FeNO}^8^ species appears best described as a resonance hybrid of Fe^I^–NO^0^ and Fe^II^–NO^–^, with a small preference for the latter. As mentioned above, a similar description was also found for the *S* = 0 {FeNO}^8^ anion ^1^{Fe[P](NO)}^−^ (P = unsubstituted porphyrin). Thus, from {FeNO}^7^ to {FeNO}^8^, the reduction appears to be substantially centered on the NO ligand.

For the {FeNO}^9^ complex ^2^{Fe(TIMEN)(NO)}, the [π*(NO)]^2^ configuration remains dominant, with the [π*(NO)]^1^ configuration accounting for only ∼30% of the wave function; the species thus seems best described as Fe^I^–NO^–^, consistent with the description of the species in the work of Meyer and co-workers.^23^ The {FeNO}^8^-to-{FeNO}^9^ reduction, according to our calculations, appears to be substantially metal-based.^23,24^ Finally, reduction to the {FeNO}^10^ state also appears to be substantially metal-centered, given that the [π*(NO)]^2^ configuration remains the dominant contributor (58.9%) to the wave function of ^1^{Fe(TIMEN)(NO)}^−^.^23,24^ We are optimistic that these findings will also extend to other {FeNO}^6–10^ systems.

#### Dinitrosyl Iron Complexes

An earlier DFT study described the DNIC ^2^{Fe[nacnac](NO)_2_} as a resonance hybrid: Fe^III^–(NO^–^)_2_ ↔ Fe^II^–(NO^–0.5^)_2_.^26^ Our DMRG-CASSCF analysis, however, indicates a somewhat different picture. We found three main configurations, with the [π*(NO)]^3^ configuration accounting for nearly half of the wave function and the remainder consisting of equal amounts of the [π*(NO)]^4^ and [π*(NO)]^2^ configurations. Thus, ^2^{Fe[nacnac](NO)_2_} seems best described as Fe^II^–(NO^–0.5^)_2_. For the superreduced DNIC species, we observed comparable contributions of the [π*(NO)]^3^ (35.7%) and [π*(NO)]^4^ (45.2%), indicating a resonance hybrid formulation for the species: Fe^II^–(NO^–^)_2_ ↔ Fe^I^–(NO^–0.5^)_2_. Thus, our analysis suggests that reduction of {Fe(NO)_2_}^9^ state involves both the metal center and the NO ligands.

### Bond Distance/Frequency Correlations

**Figure 3:**
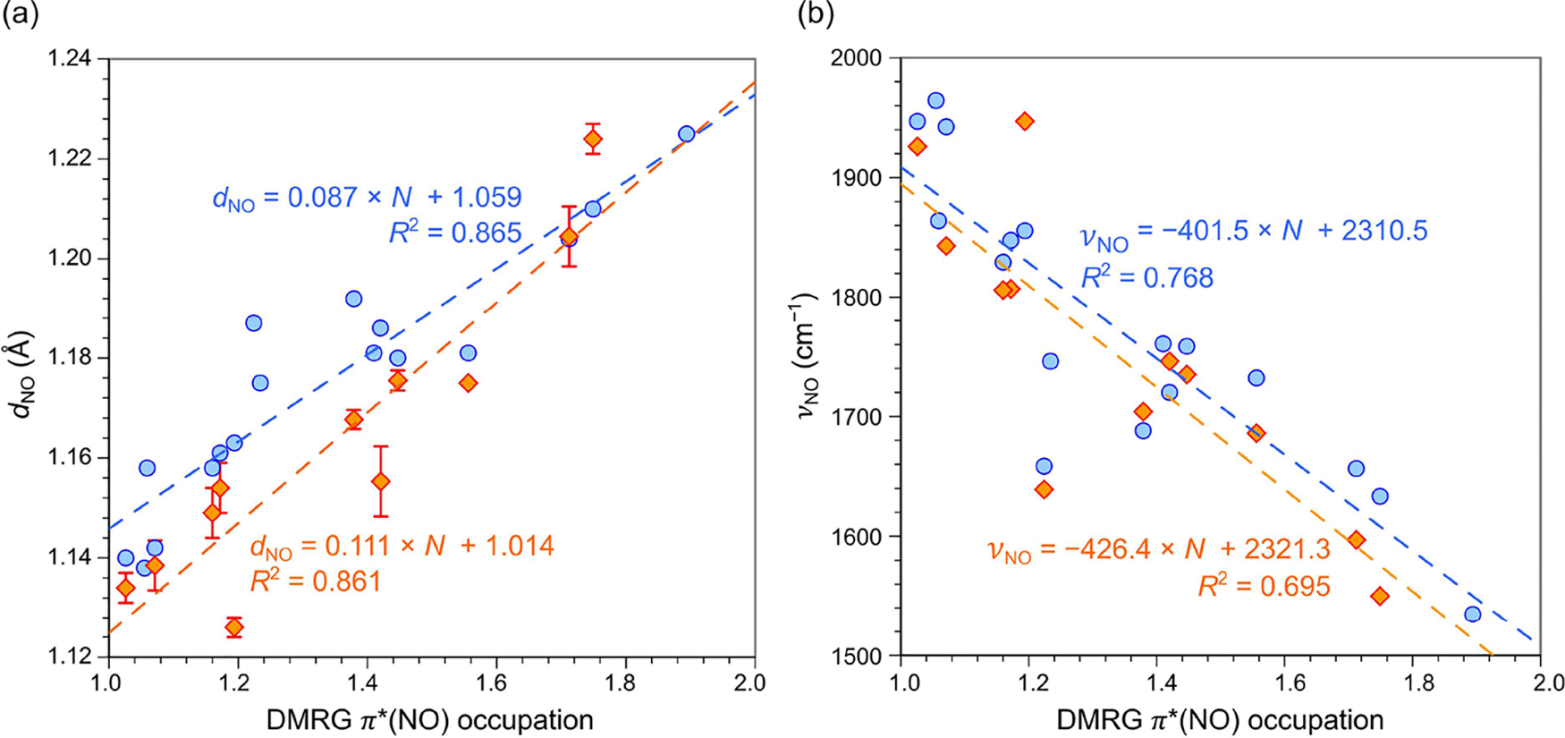
Correlation between the DMRG π*(NO) occupation number *N* and (a) the NO bond distance (*d*_NO_) and (b) the NO stretching frequency (ν_NO_). Experimental data are shown in orange, while theoretical BP86-D3(BJ) values are shown in blue. Red error bars in panel (a) represent the crystallographic uncertainties of the NO bond distance. Coefficients of determination (*R*^2^) for each data set are displayed in their respective colors.

In our earlier study of heme-nitrosyl complexes,^12^ we demonstrated the expected linear relationship among π*(NO) occupancy, the NO bond distance (*d*_NO_), and the NO stretching frequency (ν_NO_), with all quantities obtained from BP86-D3(BJ) calculations. In this study, we calculated overall π*(NO) occupation numbers (hereafter denoted *N*) using the weights *w*_*i*_ of the different configurations in DMRG-CASSCF wave functions via the expression  (note that these are the same weights that are visually depicted in Figure 2). Both experimental and BP86-D3(BJ) NO distances and frequencies were then examined as a function of *N* (Table 2 and Figures 3 and S18). As shown in Figure 3, both experimental and BP86-D3(BJ) values of *d*_NO_ exhibit an excellent linear correlation with the *N*, with an *R*^2^ value of ∼85%. For ν_NO_, the correlation is somewhat more modest, about 77% for experimental values from IR spectroscopy and about 70% for harmonic frequencies obtained from BP86-D3(BJ) calculations. The slightly lower coefficients of determination (*R*^2^) for ν_NO_ probably reflect the neglect of kinematic factors, which are expected to vary widely among the complexes. Satisfyingly, this analysis establishes a direct connection between DMRG-CASSCF results and experimental data, underscoring the overwhelming importance of the π*(NO) occupation number in determining the N–O distance and force constant for the complexes studied. Stated differently, an accurate knowledge of either *d*_NO_ and ν_NO_ (whether from experiments or DFT calculations) affords a good estimate of the overall π*(NO) occupation number and vice versa.

**Table 2:** NO Bond Distances (*d*_NO_) and Vibrational Frequencies (ν_NO_) from BP86-D3(BJ) Optimizations and Experimental Measurements as a Function of DMRG-CASSCF π*(NO) Occupation Numbers^a^

complex	source of experimental data	*d*_NO_ (Å)		ν_NO_ (cm^–1^)		DMRG π*(NO) occupation (*N*)^b^
		BP86-D3(BJ)	Exp.	BP86-D3(BJ)	Exp.	
^3^{Fe(S^*t*^Bu)(NO)}	_^c^	1.175	_^c^	1746.0	_^c^	1.234
^3^{Fe[NS3](NO)}	_^c^	1.158	_^c^	1864.0	_^c^	1.059
^3^{Fe[PS3](NO)}	(16)	1.161	1.154(5)	1847.6	1807	1.172
^1^{Fe(CN)_5_(NO)}^2–^	(21)	1.163	1.126(2)	1855.7	1947	1.194
^1^{Fe[TIMEN]-(CH_3_CN)(NO)}^3+^	(23)	1.140	1.139(3)	1947.0	1926	1.026
^4^{Fe(H_2_O)_5_(NO)}^2+^	(13)	1.142	1.139(5)^d^	1942.4	1843	1.071
^4^{Fe(H_2_O)_4_(NO)}^2+^	_^c^	1.138	_^c^	1964.4	_^c^	1.055
^4^{Fe(StBu)(NO)}^−^	(15)	1.192	1.1677(19)	1688.1	1704	1.379
^4^{Fe(NS_3_)(NO)}^−^	_^c^	1.187	_^c^	1658.5	1639	1.224
^2^{Fe(PS_3_)(NO)}^−^	_^c^	1.181	_^c^	1760.7	_^c^	1.410
^2^{Fe(CN)_4_(NO)}^2–^	(17)	1.186	1.157(7)	1720.0	1746	1.420
^4^{Fe[TIMEN](NO)}^2+^	(23)	1.158	1.149(5)	1829.3	1806	1.160
^3^{Fe[TIMEN](NO)}^+^	(23)	1.181	1.175	1732.1	1686	1.556
^2^{Fe[TIMEN](NO)}	(23)	1.210	1.224(3)	1633.4	1550	1.749
^1^{Fe[TIMEN](NO)}^−^	_^c^	1.225	_^c^	1534.6	_^c^	1.894
^2^{Fe[nacnac](NO)_2_}	(25)	1.180	1.175(2)^d^	1758.5	1735	1.447
^1^{Fe[nacnac](NO)_2_}^−^	(25)	1.204	1.204(6)	1656.5	1597	1.712

a Results for {Fe[nacnac](NO)_2_} were averaged over the two NO moieties.

b The DMRG π*(NO) occupation number (*N*) is estimated as , where *w*_*i*_ is the weight of the [π*(NO)]^*i*^ configuration.

c No experimental data or high-precision measurements are available.

d Values are averaged from two experimental results.

At a more granular level, we may observe the following:

1. Since both {FeNO}^6^ and {FeNO}^7^ complexes share a dominant [π*(NO)]^1^ configuration, it makes sense that their *d*_NO_ and ν_NO_ values span overlapping ranges. To illustrate, both experimentally and theoretically, the {FeNO}^6^ complex ^3^{Fe[PS3](NO)} and the {FeNO}^7^ complexes ^4^{Fe(H_2_O)_5_(NO)}^2+^ and ^4^{Fe[TIMEN](NO)}^2+^ share similar ν_NO_’s (which are 1800–1850 cm^–1^ for the experimental values).
2. For Enemark-Feltham counts >7, both *d*_NO_ and ν_NO_ appear to correlate better with the overall DMRG-CASSCF π*(NO) occupation number, i.e., *d*_NO_ increases monotonically and ν_NO_ decreases monotonically with the π*(NO) occupation number. Thus, the {FeNO}^8^ complex ^3^{Fe[TIMEN](NO)}^+^ and the {Fe(NO)_2_}^9^ DNIC ^2^{Fe[nacnac](NO)_2_} exhibit near-identical, longish *d*_NO_’s (1.175 Å), similar ν_NO_’s (1710 ± 25 cm^–1^), and similar DMRG-CASSCF π*(NO) occupation numbers (1.50 ± 0.06). For the {FeNO}^9^ complex ^2^{Fe[TIMEN](NO)}, the {FeNO}^10^ complex ^1^{Fe[TIMEN](NO)}^−^, and the superreduced {Fe(NO)_2_}^10^ DNIC ^1^{Fe[nacnac](NO)_2_}^−^, the π*(NO) occupation number increases significantly, with concomitantly longer *d*_NO_’s and lower ν_NO_’s (Table 2).

## Conclusions

The oxidation state of NO (i.e., NO^+^, NO^0^, NO^–^, etc.) has long been a vexatious question for transition metal nitrosyls. Following a successful DMRG-CASSCF analysis of heme-nitrosyl complexes, we have now extended the methodology to a wide range of nonheme iron nitrosyl and dinitrosyl complexes. By and large, our earlier conclusions vis-à-vis heme-nitrosyl systems, notably the broad prevalence of the NO^0^ oxidation state, appear to be eminently transferable nonheme iron systems. Although not entirely unexpected, it is nonetheless a significant finding, given that nonheme iron nitrosyls are far more diverse than their heme counterparts, in terms of the variety of ligands, coordination geometry, and spin states. Our main conclusions are as follows.

For both {FeNO}^6^ and {FeNO}^7^ complexes, the predominant resonance form corresponds to the NO^0^ oxidation state. Thus, regardless of coordination geometry and spin state (*S* = 0 or 1), the {FeNO}^6^ complexes all appear to be best described as Fe^III^–NO^0^. Notably, the often-invoked Fe^II^–NO^+^ resonance form is found to be of little importance. For {FeNO}^7^ complexes, the NO^–^ resonance form gains in importance, and indeed approaches the NO^0^ form in importance, in the presence of multiple strong-field ligands such as thiolate and cyanide.

The TIMEN (tripodal triscarbene) series of complexes sheds significant light on the site of reduction in the reduced and superreduced {FeNO}^8–10^ states. Thus, the *S* = 1 {FeNO}^8^ state appears best described as resonance hybrid: Fe^II^–NO^–^ ↔ Fe^I^–NO^0^. Further reduction to the {FeNO}^9^ state, which is best described as Fe^I^–NO^–^, thus appears to be primarily metal-centered. The most highly reduced {FeNO}^10^ state, best described as Fe^0^–NO^–^, thus also appears to arise via metal-based reduction of the {FeNO}^9^ state.

For the nacnac-supported DNICs, our analysis supports a largely Fe^II^–(NO^–0.5^)_2_ formulation for the {Fe(NO)_2_}^9^ state and an Fe^II^–(NO^–^)_2_ ↔ Fe^I^–(NO^–0.5^)_2_ resonance hybrid for the {FeNO}^10^ state. In other words, reduction of the {Fe(NO)_2_}^9^ unit, according to our analysis, involves not only the metal center but also the NO ligands, a finding somewhat at odds with literature favoring primarily metal-based reduction.

In this study, we used the weights of the [π*(NO)]^*i*^ (*i* = 1–4 for mononitrosyl complexes) resonance forms to derive an overall DMRG-CASSCF-based π*(NO) occupation number *N*, which was found to exhibit a linear correlation with N–O bond distances and stretching frequencies. In other words, a theoretical knowledge of *N* allows an effective prediction of NO distances and force constants. By the same token, an accurate experimental NO distance or an NO stretching frequency allows us to simply use our plots to read off an estimated π*(NO) occupation number and hence a local oxidation state for the NO unit.

Finally, and fascinatingly, in spite of the distinctly post-Enemark-Feltham spirit of this study, where we have sought to define the local oxidation states of iron-bound NO units, the Enemark-Feltham formalism retains considerable relevance. A careful reading of the above results should convince the reader that there is actually a one-to-one mapping between the Enemark-Feltham count and local oxidation states. This mapping (which is graphically depicted as part of the abstract) also holds for heme-nitrosyl species, which we examined in our earlier study.^12^ However, at present we view the mapping as specific to iron nitrosyls; exactly how the mapping might change as a function of the metal remains the subject of future studies.

## Data Availability

All data generated or analyzed in this study are included in this published article and its Supporting Information.

## Supporting Information Available

Mulliken spin population values calculated with different exchange-correlation functionals and DMRG-CASSCF theory, active orbitals in DMRG-CASSCF calculations, and DFT and DMRG-CASSCF total energies (PDF)
Optimized Cartesian coordinates of the complexes (ZIP)
