# Supplementary material for: NO Oxidation States in Nonheme Iron Nitrosyls: A DMRG-CASSCF Study of {FeNO}6–10 Complexes
Source: Inorg Chem. 2025 Jan 23;64(4):1702–10. doi: 10.1021/acs.inorgchem.4c03845 (PMC11795527; doi:10.1021/acs.inorgchem.4c03845)
Supplement: Supplementary file 1 — ic4c03845_si_001.pdf [file ic4c03845_si_001.pdf]

Supporting Information

NO Oxidation States in Nonheme Iron Nitrosyls: A DMRG-CASSCF Study of $\{FeNO\}^{6–10}$ Complexes

Quan Manh Phung,[*,†,‡] Ho Ngoc Nam,[¶] Vic Austen,[†] Takeshi Yanai,[†,‡] and Abhik Ghosh[*,§]

†*Department of Chemistry, Graduate School of Science, Nagoya University, Furo-cho, Chikusa-ku, Nagoya, Aichi 464-8602, Japan*

‡*Institute of Transformative Bio-Molecules (WPI-ITbM), Nagoya University, Furo-cho, Chikusa-ku, Nagoya, Aichi 464-8601, Japan*

¶*Department of Materials Process Engineering, Graduate School of Engineering, Nagoya University, Furo-cho, Chikusa-ku, Nagoya, Aichi 464-8603, Japan*

§*Department of Chemistry, UiT The Arctic University of Norway, N09037 Tromsø, Norway*

E-mail: quan.phung@chem.nagoya-u.ac.jp; abhik.ghosh@uit.no

This document contains the raw energetic data, spin populations of atoms, and geometric data of the investigated species, along with their active natural orbitals in the DMRG-CASSCF calculations.

List of Figures

S1 Representations of the 18 active natural orbitals in the DMRG-CASSCF calculation of the $^4\{Fe(H_2O)_5(NO)\}^{2+}$ complex (quartet state) with isovalue = 0.04. The number next to each orbital represents the occupation number . . S14

S2 Representations of the 18 active natural orbitals in the DMRG-CASSCF calculations of the $^4\{Fe(H_2O)_4(NO)\}^{2+}$ complex (quartet state) with isovalue = 0.04. The number next to each orbital represents the occupation number . . S17

S3 Representations of the 18 active natural orbitals in the DMRG-CASSCF calculations of the $^3\{Fe(S^tBu)_3(NO)\}$ complex (triplet state) with isovalue = 0.04. The number next to each orbital represents the occupation number . . S20

S4 Representations of the 18 active natural orbitals in the DMRG-CASSCF calculations of the $^4\{Fe(S^tBu)_3(NO)\}^-$ complex (quartet state) with isovalue = 0.04. The number next to each orbital represents the occupation number . . S23

S5 Representations of the 20 active natural orbitals in the DMRG-CASSCF calculations of the $^3\{Fe[NS3](NO)\}$ complex (triplet state) with isovalue = 0.04. The number next to each orbital represents the occupation number S26

S6 Representations of the 18 active natural orbitals in the DMRG-CASSCF calculations of the $^4\{Fe[NS3](NO)\}^-$ complex (quartet state) with isovalue = 0.04. The number next to each orbital represents the occupation number . . S29

S7 Representations of the 20 active natural orbitals in the DMRG-CASSCF calculations of the $^3\{Fe[PS3](NO)\}$ complex (triplet state) with isovalue = 0.04. The number next to each orbital represents the occupation number S32

S8 Representations of the 18 active natural orbitals in the DMRG-CASSCF calculations of the $^2\{Fe[PS3](NO)\}^-$ complex (doublet state) with isovalue = 0.04. The number next to each orbital represents the occupation number . . S35

S9 Representations of the 18 active natural orbitals in the DMRG-CASSCF calculations of the ${}^{1}\{Fe(CN)_5(NO)\}^{2-}$ complex (singlet state) with isovalue = 0.04. The number next to each orbital represents the occupation number . . S38
S10 Representations of the 18 active natural orbitals in the DMRG-CASSCF calculations of the ${}^{2}\{Fe(CN)_4(NO)\}^{2-}$ complex (doublet state) with isovalue = 0.04. The number next to each orbital represents the occupation number . . S41
S11 Representations of the 18 active natural orbitals in the DMRG-CASSCF calculations of the ${}^{1}\{Fe[TIMEN](CH_3CN)(NO)\}^{3+}$ complex (singlet state) with isovalue = 0.04. The number next to each orbital represents the occupation number S44
S12 Representations of the 18 active natural orbitals in the DMRG-CASSCF calculations of the ${}^{4}\{Fe[TIMEN](NO)\}^{2+}$ complex (quartet state) with isovalue = 0.04. The number next to each orbital represents the occupation number . S47
S13 Representations of the 18 active natural orbitals in the DMRG-CASSCF calculations of the ${}^{3}\{Fe[TIMEN](NO)\}^{+}$ complex (triplet state) with isovalue = 0.04. The number next to each orbital represents the occupation number . . S50
S14 Representations of the 18 active natural orbitals in the DMRG-CASSCF calculations of the ${}^{2}\{Fe[TIMEN](NO)\}$ complex (doublet state) with isovalue = 0.04. The number next to each orbital represents the occupation number . . S52
S15 Representations of the 18 active natural orbitals in the DMRG-CASSCF calculations of the ${}^{1}\{Fe[TIMEN](NO)\}^{-}$ complex (singlet state) with isovalue = 0.04. The number next to each orbital represents the occupation number . . S54
S16 Representations of the 25 active natural orbitals in the DMRG-CASSCF calculations of the ${}^{2}\{Fe[nacnac](NO)_2\}$ complex (doublet state) with isovalue = 0.04. The number next to each orbital represents the occupation number . . S57

S17 Representations of the 25 active natural orbitals in the DMRG-CASSCF calculations of the $^{1}\{Fe[nacnac](NO)_2\}^{-}$ complex (singlet state) with isovalue = 0.04. The number next to each orbital represents the occupation number . . S59
S18 Correlation between NO bond distance and vibrational frequency, calculated at the BP86-D3(BJ)/def2-TZVP level of theory. The orange line is the calibration curve, obtained by a linear fit to the frequencies of isolated NO molecules with different NO(π^*) occupancies, represented by open circles. S60

List of Tables

S1 Description of active space of the complexes studied in this work S10
S2 Electronic energies E (in E_h) and spin populations for Fe, N, O, and NO for $^{4}\{Fe(H_2O)_5(NO)\}^{2+}$ (quartet state), calculated using different DFT functionals and DMRG-CASSCF S11
S3 Electronic energies E (in E_h) and spin populations for Fe, N, O, and NO for $^{2}\{Fe(H_2O)_5(NO)\}^{2+}$ (doublet state), calculated using different DFT functionals S11
S4 Energy difference (in kcal/mol) between low-spin (doublet) and high-spin (quartet) states of $\{Fe(H_2O)_5(NO)\}^{2+}$, calculated using different DFT functionals S12
S5 Geometric data for $^{4}\{Fe(H_2O)_5(NO)\}^{2+}$, calculated with BP86 . . . . S12
S6 Weights (in percentage) of dominant configurations based on $[\pi^*(NO)]^n$ (n = 0, 1, 2, 3) occupancy in DMRG-CASSCF wave functions in $^{4}\{Fe(H_2O)_5(NO)\}^{2+}$, using different localization methods S12
S7 Selected bond distances (in Å) and Fe−NO bond angle (in degree) in $^{4}\{Fe(H_2O)_5(NO)\}^{2+}$, calculated with different DFT functionals S12

S8 Weights (in percentage) of dominant configurations based on $[\pi^*(\mathrm{NO})]^n$ ($n =$ 0, 1, 2, 3) occupancy in DMRG-CASSCF wave functions in $^4\{\mathrm{Fe(H_2O)_5(NO)}\}^{2+}$, using BP86, TPSSh, B3LYP, and PBE0 optimized structures S12
S9 Comparison between DMRG-CASSCF results with $m = 1000$ and 2000 in $^4\{\mathrm{Fe(H_2O)_5(NO)}\}^{2+}$ S13
S10 Electronic energies E (in E_h) and spin populations for Fe, N, O, and NO for $^4\{\mathrm{Fe(H_2O)_4(NO)}\}^{2+}$ (quartet state), calculated using different DFT functionals and DMRG-CASSCF S15
S11 Electronic energies E (in E_h) and spin populations for Fe, N, O, and NO for $^2\{\mathrm{Fe(H_2O)_4(NO)}\}^{2+}$ (doublet state), calculated using different DFT functionals S15
S12 Energy difference between low-spin (doublet) and high-spin (quartet) states of $\{\mathrm{Fe(H_2O)_4(NO)}\}^{2+}$, calculated using different DFT functionals S16
S13 Geometric data for $^2\{\mathrm{Fe(H_2O)_4(NO)}\}^{2+}$, calculated with BP86 . . . . S16
S14 Electronic energies E (in E_h) and spin populations for Fe, N, O and, NO for $^3\{\mathrm{Fe(S^tBu)_3(NO)}\}$ (triplet state), calculated using different DFT functionals and DMRG-CASSCF S18
S15 Electronic energies E (in E_h) and spin populations for Fe, N, O, and NO for $^1\{\mathrm{Fe(S^tBu)_3(NO)}\}$ (singlet state), calculated using different DFT functionals S18
S16 Energy difference between low-spin (singlet) and high-spin (triplet) states of $\{\mathrm{Fe(S^tBu)_3(NO)}\}$, calculated using different DFT functionals S19
S17 Geometric data for $^3\{\mathrm{Fe(S^tBu)_3(NO)}\}$, calculated with BP86 . . . . S19
S18 Electronic energies E (in E_h) and spin populations for Fe, N, O, and NO for $^4\{\mathrm{Fe(S^tBu)_3(NO)}\}^-$ (quartet state), calculated using different DFT functionals and DMRG-CASSCF S21
S19 Electronic energies E (in E_h) and spin populations for Fe, N, O, and NO for $^2\{\mathrm{Fe(S^tBu)_3(NO)}\}^-$ (doublet state), calculated using different DFT functionals S21

S20 Energy difference between low-spin (doublet) and high-spin (quartet) states of $\{Fe(S^tBu)_3(NO)\}^-$, calculated using different DFT functionals S22
S21 Geometric data for $^4\{Fe(S^tBu)_3(NO)\}^-$, calculated with BP86 . . . . S22
S22 Electronic energies E (in E_h) and spin populations for Fe, N, O, and NO for $^3\{Fe[NS3](NO)\}$ (triplet state), calculated using different DFT functionals and DMRG-CASSCF S24
S23 Electronic energies E (in E_h) and spin populations for Fe, N, O, and NO for $^1\{Fe[NS3](NO)\}$ (singlet state), calculated using different DFT functionals . S24
S24 Energy difference between low-spin (singlet) and high-spin (triplet) states of $\{Fe[NS3](NO)\}$, calculated using different DFT functionals S25
S25 Geometric data for $^3\{Fe[NS3](NO)\}$, calculated using BP86 . . . . S25
S26 Electronic energies E (in E_h) and spin populations for Fe, N, O, and NO for $^4\{Fe[NS3](NO)\}^-$ (quartet state), calculated using different DFT functionals and DMRG-CASSCF S27
S27 Electronic energies E (in E_h) and spin populations for Fe, N, O, and NO for $^2\{Fe[NS3](NO)\}^-$ (doublet state), calculated using different DFT functionals S27
S28 Energy difference between low-spin (doublet) and high-spin (quartet) states of $\{Fe[NS3](NO)\}^-$, calculated using different DFT functionals S28
S29 Geometric data for $^4\{Fe[NS3](NO)\}^-$, calculated with BP86 . . . . S28
S30 Electronic energies E (in E_h) and spin populations for Fe, N, O, and NO for $^3\{Fe[PS3](NO)\}$ (triplet state), calculated using different DFT functionals and DMRG-CASSCF S30
S31 Electronic energies E (in E_h) and spin populations for Fe, N, O, and NO for $^1\{Fe[PS3](NO)\}$ (singlet state), calculated using different DFT functionals . S30
S32 Energy difference between low-spin (singlet) and high-spin (triplet) states of $\{Fe[PS3](NO)\}$, calculated using different DFT functionals S31
S33 Geometric data for $^3\{Fe[PS3](NO)\}$, calculated with BP86 . . . . S31

S34 Electronic energies E (in E_h) and spin populations for Fe, N, O, and NO for $^4\{Fe[PS3](NO)\}^-$ (quartet state), calculated using different DFT functionals . S33
S35 Electronic energies E (in E_h) and spin populations for Fe, N, O, and NO for $^2\{Fe[PS3](NO)\}^-$ (doublet state), calculated using different DFT functionals and DMRG-CASSCF S33
S36 Energy difference between low-spin (doublet) and high-spin (quartet) states of $\{Fe[PS3](NO)\}^-$, calculated using different DFT functionals S34
S37 Geometric data for $^2\{Fe[PS3](NO)\}^-$, calculated with BP86 . . . . S34
S38 Electronic energies E (in E_h) and spin populations for Fe, N, O and, NO for $^3\{Fe(CN)_5(NO)\}^{2-}$ (triplet state), calculated using different DFT functionals S36
S39 Electronic energies E (in E_h) and spin populations for Fe, N, O, and NO for $^1\{Fe(CN)_5(NO)\}^{2-}$ (singlet state), calculated using different DFT functionals and DMRG-CASSCF S36
S40 Energy difference between low-spin (singlet) and high-spin (triplet) states of $\{Fe(CN)_5(NO)\}^{2-}$, calculated using different DFT functionals S37
S41 Geometric data for $^1\{Fe(CN)_5(NO)\}^{2-}$, calculated with BP86 . . . . S37
S42 Electronic energies E (in E_h) and spin populations for Fe, N, O, and NO for $^4\{Fe(CN)_4(NO)\}^{2-}$ (quartet state), calculated using different DFT functionals S39
S43 Electronic energies E (in E_h) and spin populations for Fe, N, O, and NO for $^2\{Fe(CN)_4(NO)\}^{2-}$ (doublet state), calculated using different DFT functionals and DMRG-CASSCF S39
S44 Energy difference between low-spin (doublet) and high-spin (quartet) states of $\{Fe(CN)_4(NO)\}^{2-}$, calculated using different DFT functionals S40
S45 Geometric data for $^2\{Fe(CN)_4(NO)\}^{2-}$, calculated using BP86 . . . . S40
S46 Electronic energies E (in E_h) and spin populations for Fe, N, O, and NO for $^3\{Fe[TIMEN](CH_3CN)(NO)\}^{3+}$ (triplet state), calculated using different DFT functionals S42

S47 Electronic energies E (in E_h) and spin populations for Fe, N, O, and NO for $^1\{Fe[TIMEN](CH_3CN)(NO)\}^{3+}$ (singlet state), calculated using different DFT functionals and DMRG-CASSCF S42
S48 Energy difference between low-spin (singlet) and high-spin (triplet) states of $\{Fe[TIMEN](CH_3CN)(NO)\}^{3+}$, calculated using different DFT functionals . . S43
S49 Geometric data for $^1\{Fe[TIMEN](CH_3CN)(NO)\}^{3+}$, calculated with BP86 . . S43
S50 Electronic energies E (in E_h) and spin populations for Fe, N, O, and NO for $^4\{Fe[TIMEN](NO)\}^{2+}$ (quartet state), calculated using different DFT functionals and DMRG-CASSCF S45
S51 Electronic energies E (in E_h) and spin populations for Fe, N, O, and NO for $^2\{Fe[TIMEN](NO)\}^{2+}$ (doublet state), calculated using different DFT functionals S45
S52 Energy difference between low-spin (doublet) and high-spin (quartet) states of $\{Fe[TIMEN](NO)\}^{2+}$, calculated using different DFT functionals S46
S53 Geometric data for $^4\{Fe[TIMEN](NO)\}^{2+}$, calculated with BP86 . . . . S46
S54 Electronic energies E (in E_h) and spin populations for Fe, N, O, and NO for $^3\{Fe[TIMEN](NO)\}^{+}$ (triplet state), calculated using different DFT functionals and DMRG-CASSCF S48
S55 Electronic energies E (in E_h) and spin populations for Fe, N, O, and NO for $^1\{Fe[TIMEN](NO)\}^{+}$ (singlet state), calculated using different DFT functionalsS48
S56 Energy difference between low-spin (singlet) and high-spin (triplet) states of $\{Fe[TIMEN](NO)\}^{+}$, calculated using different DFT functionals S49
S57 Geometric data for $^3\{Fe[TIMEN](NO)\}^{+}$, calculated using BP86 . . . . S49
S58 Electronic energies E (in E_h) and spin populations for Fe, N, O, and NO for $^2\{Fe[TIMEN](NO)\}$ (doublet state), calculated using different DFT functionals and DMRG-CASSCF S51
S59 Geometric data for $^2\{Fe[TIMEN](NO)\}$, calculated with BP86 . . . . S51

S60 Electronic energies E (in E_h) and spin populations for Fe, N, O, and NO for $^1\{Fe[TIMEN](NO)\}^-$ (singlet state), calculated using different DFT functionals and DMRG-CASSCF S53
S61 Geometric data for $^1\{Fe[TIMEN](NO)\}^-$, calculated with BP86 . . . . S53
S62 Electronic energies E (in E_h) and spin populations for Fe, N, O, and NO for $^2\{Fe[nacnac](NO)_2\}$ (doublet state), calculated using different DFT functionals and DMRG-CASSCF S55
S63 Geometric data for $^2\{Fe[nacnac](NO)_2\}$, calculated using BP86 . . . . S55
S64 Comparison between DMRG-CASSCF results with $m = 1000$ and 1500 in $^2\{Fe[nacnac](NO)_2\}$ S56
S65 Electronic energies E (in E_h) and spin populations for Fe, N, O, and NO for $^1\{Fe[nacnac](NO)_2\}^-$ (singlet state), calculated using different DFT functionals and DMRG-CASSCF S58
S66 Geometric data for $^1\{Fe[nacnac](NO)_2\}^-$, calculated with BP86. . . . . S58

Table S1: Description of active space of the complexes studied in this work

Complex	Active space	Number of Fe(4d) orbitals
$^{4}\{Fe(H_2O)_5(NO)\}^{2+}$	CAS(13,18)	5
$^{4}\{Fe(H_2O)_4(NO)\}^{2+}$	CAS(13,18)	5
$^{3}\{Fe(S^tBu)_3(NO)\}$	CAS(14,18)	4
$^{4}\{Fe(S^tBu)_3(NO)\}^{-}$	CAS(13,18)	5
$^{3}\{Fe[NS3](NO)\}$	CAS(18,20)	4
$^{4}\{Fe[NS3](NO)\}^{-}$	CAS(13,18)	5
$^{3}\{Fe[PS3](NO)\}$	CAS(18,20)	4
$^{2}\{Fe[PS3](NO)\}^{-}$	CAS(13,18)	4
$^{1}\{Fe(CN)_5(NO)\}^{2-}$	CAS(16,18)	3
$^{2}\{Fe(CN)_4(NO)\}^{2-}$	CAS(15,18)	4
$^{1}\{Fe[TIMEN](CH_3CN)(NO)\}^{3+}$	CAS(16,18)	3
$^{4}\{Fe[TIMEN](NO)\}^{2+}$	CAS(13,18)	5
$^{3}\{Fe[TIMEN](NO)\}^{+}$	CAS(14,18)	5
$^{2}\{Fe[TIMEN](NO)\}$	CAS(15,18)	5
$^{1}\{Fe[TIMEN](NO)\}^{-}$	CAS(16,18)	5
$^{2}\{Fe[nacnac](NO)_2\}$	CAS(21,25)	4
$^{1}\{Fe[nacnac](NO)_2\}^{-}$	CAS(22,25)	4

Results for $\{Fe(H_2O)_5(NO)\}^{2+}$

Table S2: Electronic energies E (in E_h) and spin populations for Fe, N, O, and NO for $^4\{Fe(H_2O)_5(NO)\}^{2+}$ (quartet state), calculated using different DFT functionals and DMRG-CASSCF

Method	Energy (E_h)	Spin Population			
		Fe	N	O	NO
B3LYP	-1775.260872	3.760	-0.424	-0.512	-0.936
B97-D	-1775.784360	3.585	-0.392	-0.414	-0.807
BP86	-1775.826296	3.495	-0.351	-0.356	-0.707
M06-L	-1775.440588	3.716	-0.421	-0.469	-0.890
M06	-1775.250363	3.789	-0.441	-0.527	-0.968
PBE0	-1774.778203	3.844	-0.440	-0.552	-0.992
PBE	-1774.796995	3.486	-0.348	-0.355	-0.703
TPSSh	-1775.564393	3.697	-0.414	-0.454	-0.868
TPSS	-1775.658903	3.523	-0.360	-0.363	-0.724
DMRG-CASSCF	-1771.858007	3.441	-0.261	-0.224	-0.485

Table S3: Electronic energies E (in E_h) and spin populations for Fe, N, O, and NO for $^2\{Fe(H_2O)_5(NO)\}^{2+}$ (doublet state), calculated using different DFT functionals

Method	Energy (E_h)	Spin Population			
		Fe	N	O	NO
B3LYP	-1775.217442	1.768	-0.373	-0.424	-0.797
B97-D	-1775.742301	1.461	-0.250	-0.238	-0.488
BP86	-1775.804792	1.281	-0.165	-0.161	-0.327
M06-L	-1775.392710	1.499	-0.246	-0.269	-0.515
M06	-1775.186130	1.775	-0.374	-0.425	-0.799
PBE0	-1774.728490	1.880	-0.414	-0.487	-0.900
PBE	-1774.774974	1.275	-0.164	-0.159	-0.324
TPSSh	-1775.532075	1.580	-0.297	-0.311	-0.608
TPSS	-1775.638559	1.297	-0.171	-0.167	-0.337

Table S4: Energy difference (in kcal/mol) between low-spin (doublet) and high-spin (quartet) states of $\{Fe(H_2O)_5(NO)\}^{2+}$, calculated using different DFT functionals

Functional	ΔE(LS-HS)
B3LYP	27.3
B97-D	26.4
BP86	13.5
M06-L	30.0
M06	40.3
PBE0	31.2
PBE	13.8
TPSSh	20.3
TPSS	12.8

Table S5: Geometric data for $^4\{Fe(H_2O)_5(NO)\}^{2+}$, calculated with BP86

d(Fe-N) (Å)	d(N-O) (Å)	ν(N-O) (cm^{-1})
1.759	1.142	1942.42

Table S6: Weights (in percentage) of dominant configurations based on $[\pi^*(NO)]^n$ ($n = 0$, 1, 2, 3) occupancy in DMRG-CASSCF wave functions in $^4\{Fe(H_2O)_5(NO)\}^{2+}$, using different localization methods

Method	$[\pi^*(NO)]^0$	$[\pi^*(NO)]^1$	$[\pi^*(NO)]^2$	$[\pi^*(NO)]^3$
Pikek–Mezey	7.1	78.2	14.3	0.1
Boys	4.7	75.7	19.1	0.2
Edmiston–Ruedenberg	4.1	74.7	20.6	0.3

Table S7: Selected bond distances (in Å) and Fe−NO bond angle (in degree) in $^4\{Fe(H_2O)_5(NO)\}^{2+}$, calculated with different DFT functionals

Method	d(Fe–NO) (Å)	d(N–O) (Å)	∠Fe–NO (degree)
BP86	1.759	1.142	179.83
TPSSh	1.779	1.134	179.69
B3LYP	1.799	1.131	179.80
PBE0	1.786	1.128	179.58

Table S8: Weights (in percentage) of dominant configurations based on $[\pi^*(NO)]^n$ ($n = 0$, 1, 2, 3) occupancy in DMRG-CASSCF wave functions in $^4\{Fe(H_2O)_5(NO)\}^{2+}$, using BP86, TPSSh, B3LYP, and PBE0 optimized structures

Method	$[\pi^*(NO)]^0$	$[\pi^*(NO)]^1$	$[\pi^*(NO)]^2$	$[\pi^*(NO)]^3$
BP86	7.1	78.2	14.3	0.1
TPSSh	7.0	79.5	13.2	0.0
B3LYP	7.0	80.9	11.9	0.0
PBE0	7.1	80.0	12.7	0.0

Table S9: Comparison between DMRG-CASSCF results with $m = 1000$ and 2000 in $^4\{Fe(H_2O)_5(NO)\}^{2+}$

	$m = 1000$	$m = 2000$
Energy(E_h)^a	−1771.858007	−1771.858021
Energy(E_h)^b	−1771.858024	−
Weights (in percentage) of configurations		
$[\pi^*(NO)]^0$	7.116	7.118
$[\pi^*(NO)]^1$	78.226	78.224
$[\pi^*(NO)]^2$	14.310	14.311
$[\pi^*(NO)]^3$	0.060	0.060

[a] Energies calculated with CheMPS2, natural orbitals, Fiedler ordering.
[b] Energies calculated with BLOCK2, localized orbitals, Fiedler ordering.

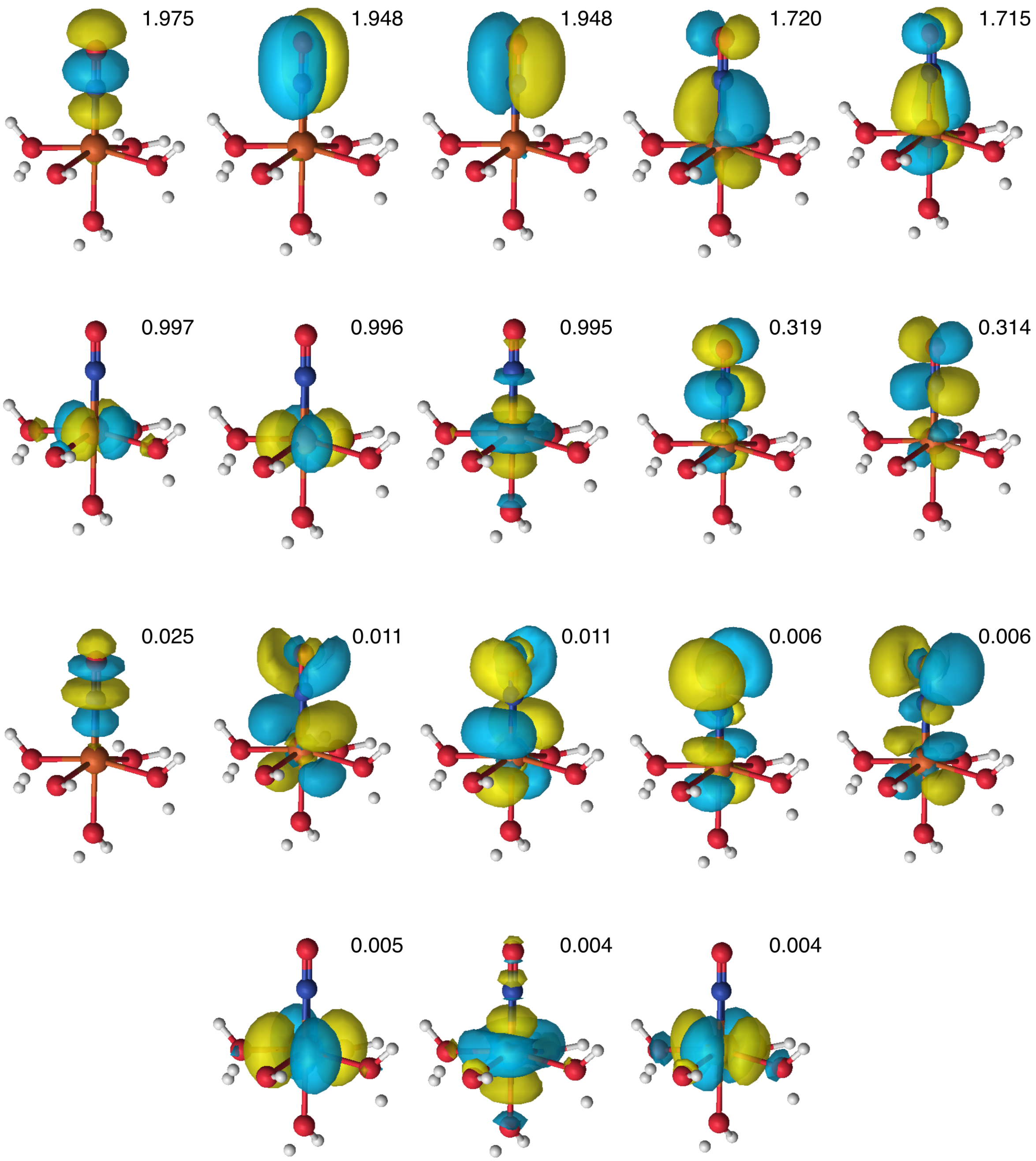

Figure S1: Representations of the 18 active natural orbitals in the DMRG-CASSCF calculation of the $^4\{Fe(H_2O)_5(NO)\}^{2+}$ complex (quartet state) with isovalue = 0.04. The number next to each orbital represents the occupation number

Results for $\{Fe(H_2O)_4(NO)\}^{2+}$

Table S10: Electronic energies E (in E_h) and spin populations for Fe, N, O, and NO for $^4\{Fe(H_2O)_4(NO)\}^{2+}$ (quartet state), calculated using different DFT functionals and DMRG-CASSCF

Method	Energy (E_h)	Spin Population			
		Fe	N	O	NO
B3LYP	-1698.788736	3.693	-0.370	-0.493	-0.863
B97-D	-1699.318185	3.510	-0.332	-0.394	-0.726
BP86	-1699.314901	3.432	-0.297	-0.335	-0.632
M06-L	-1698.949610	3.664	-0.367	-0.447	-0.814
M06	-1698.778721	3.741	-0.385	-0.511	-0.896
PBE0	-1698.354619	3.781	-0.388	-0.533	-0.921
PBE	-1698.375194	3.422	-0.293	-0.335	-0.628
TPSSh	-1699.063101	3.627	-0.356	-0.430	-0.787
TPSS	-1699.149815	3.455	-0.303	-0.340	-0.644
DMRG-CASSCF	-1695.760575	3.417	-0.235	-0.222	-0.457

Table S11: Electronic energies E (in E_h) and spin populations for Fe, N, O, and NO for $^2\{Fe(H_2O)_4(NO)\}^{2+}$ (doublet state), calculated using different DFT functionals

Method	Energy (E_h)	Spin Population			
		Fe	N	O	NO
B3LYP	-1698.743643	1.610	-0.244	-0.365	-0.610
B97-D	-1699.273066	1.344	-0.129	-0.196	-0.325
BP86	-1699.290625	1.182	-0.050	-0.118	-0.167
M06-L	-1698.899995	1.366	-0.138	-0.211	-0.348
M06	-1698.713445	1.684	-0.291	-0.401	-0.692
PBE0	-1698.302882	1.721	-0.285	-0.429	-0.713
PBE	-1698.350169	1.176	-0.048	-0.119	-0.166
TPSSh	-1699.029408	1.359	-0.133	-0.210	-0.343
TPSS	-1699.127782	1.160	-0.039	-0.104	-0.143

Table S12: Energy difference between low-spin (doublet) and high-spin (quartet) states of $\{Fe(H_2O)_4(NO)\}^{2+}$, calculated using different DFT functionals

Functional	ΔE(LS-HS)
B3LYP	27.3
B97-D	26.4
BP86	13.5
M06-L	30
M06	40.3
PBE0	31.2
PBE0	13.8
TPSSh	20.3
TPSS	12.8

Table S13: Geometric data for $^2\{Fe(H_2O)_4(NO)\}^{2+}$, calculated with BP86

d(Fe-N) (Å)	d(N-O) (Å)	ν(N-O) (cm^{-1})
1.749	1.138	1964.41

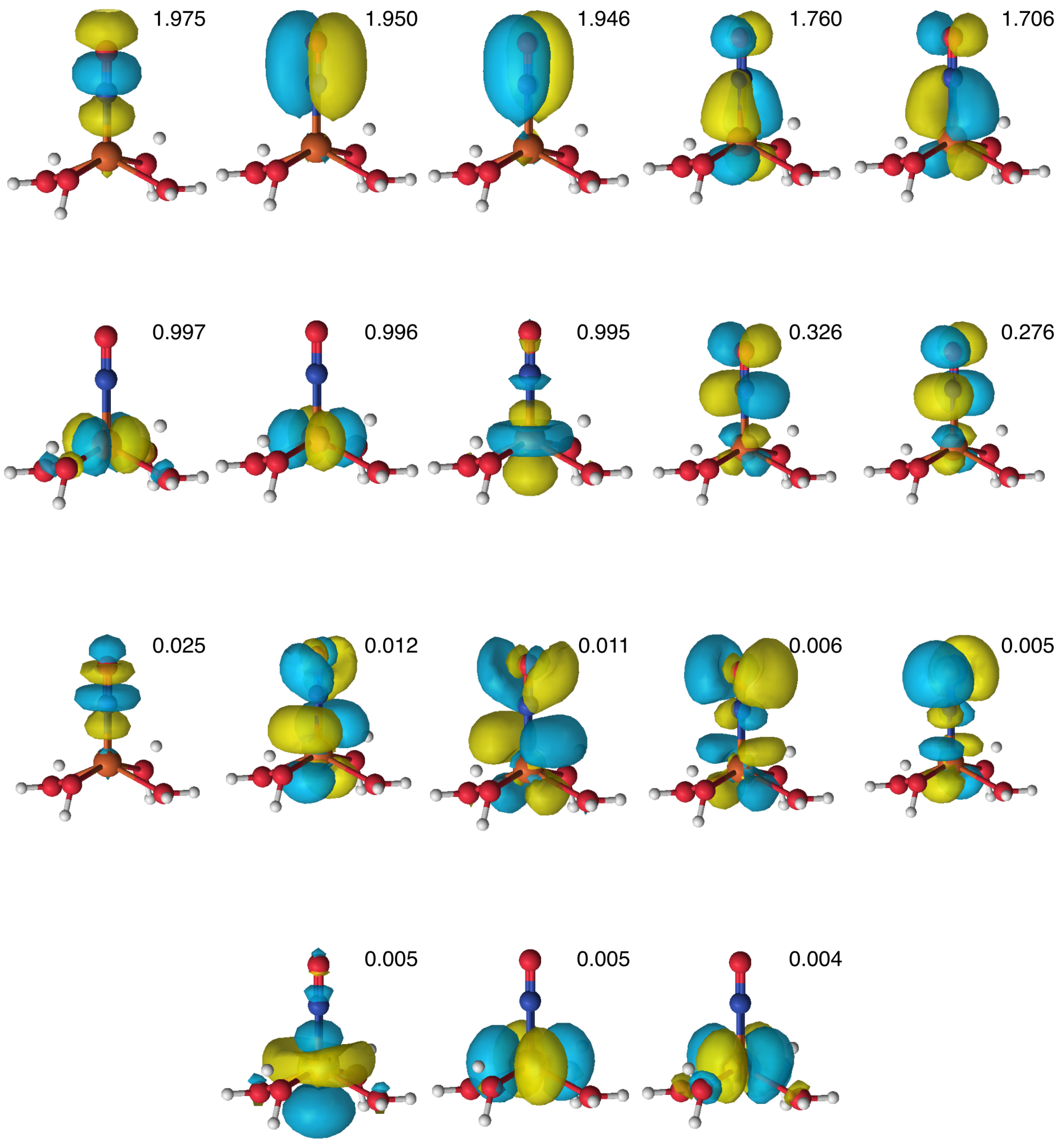

Figure S2: Representations of the 18 active natural orbitals in the DMRG-CASSCF calculations of the ${}^4\{Fe(H_2O)_4(NO)\}^{2+}$ complex (quartet state) with isovalue = 0.04. The number next to each orbital represents the occupation number

Results for $\{Fe(S^tBu)_3(NO)\}$

Table S14: Electronic energies E (in E_h) and spin populations for Fe, N, O and, NO for $^3\{Fe(S^tBu)_3(NO)\}$ (triplet state), calculated using different DFT functionals and DMRG-CASSCF

Method	Energy (E_h)	Spin Population			
		Fe	N	O	NO
B3LYP	-3061.602451	2.504	-0.386	-0.370	-0.756
B97-D	-3062.364492	1.858	-0.164	-0.158	-0.322
BP86	-3062.668488	1.559	-0.057	-0.072	-0.129
M06-L	-3062.019181	2.224	-0.261	-0.271	-0.532
M06	-3061.572786	2.753	-0.458	-0.448	-0.906
PBE0	-3060.786889	2.845	-0.475	-0.457	-0.932
PBE	-3060.692303	1.560	-0.056	-0.072	-0.128
TPSSh	-3062.330376	2.082	-0.223	-0.213	-0.436
TPSS	-3062.473090	1.604	-0.059	-0.072	-0.130
DMRG-CASSCF	-3054.867252	2.378	-0.167	-0.141	-0.308

Table S15: Electronic energies E (in E_h) and spin populations for Fe, N, O, and NO for $^1\{Fe(S^tBu)_3(NO)\}$ (singlet state), calculated using different DFT functionals

Method	Energy (E_h)	Spin Population			
		Fe	N	O	NO
B3LYP	-3061.583648	0.343	-0.078	-0.110	-0.188
B97-D	-3062.350903	0.205	-0.049	-0.070	-0.118
BP86	-3062.659808	0.167	-0.027	-0.054	-0.081
M06-L	-3061.999074	0.298	-0.048	-0.080	-0.127
M06	-3061.539626	1.078	-0.362	-0.369	-0.731
PBE0	-3060.761052	0.414	-0.093	-0.130	-0.223
PBE	-3060.683428	0.166	-0.027	-0.054	-0.081
TPSSh	-3062.318068	0.212	-0.022	-0.056	-0.078
TPSS	-3062.464468	0.166	-0.016	-0.045	-0.061

Table S16: Energy difference between low-spin (singlet) and high-spin (triplet) states of $\{Fe(S^tBu)_3(NO)\}$, calculated using different DFT functionals

Functional	ΔE(LS-HS)
B3LYP	11.8
B97-D	8.5
BP86	5.4
M06-L	12.6
M06	20.8
PBE0	16.2
PBE0	5.6
TPSSh	7.7
TPSS	5.4

Table S17: Geometric data for $^3\{Fe(S^tBu)_3(NO)\}$, calculated with BP86

d(Fe-N) (Å)	d(N-O) (Å)	ν(N-O) (cm^{-1})
1.67	1.175	1745.96

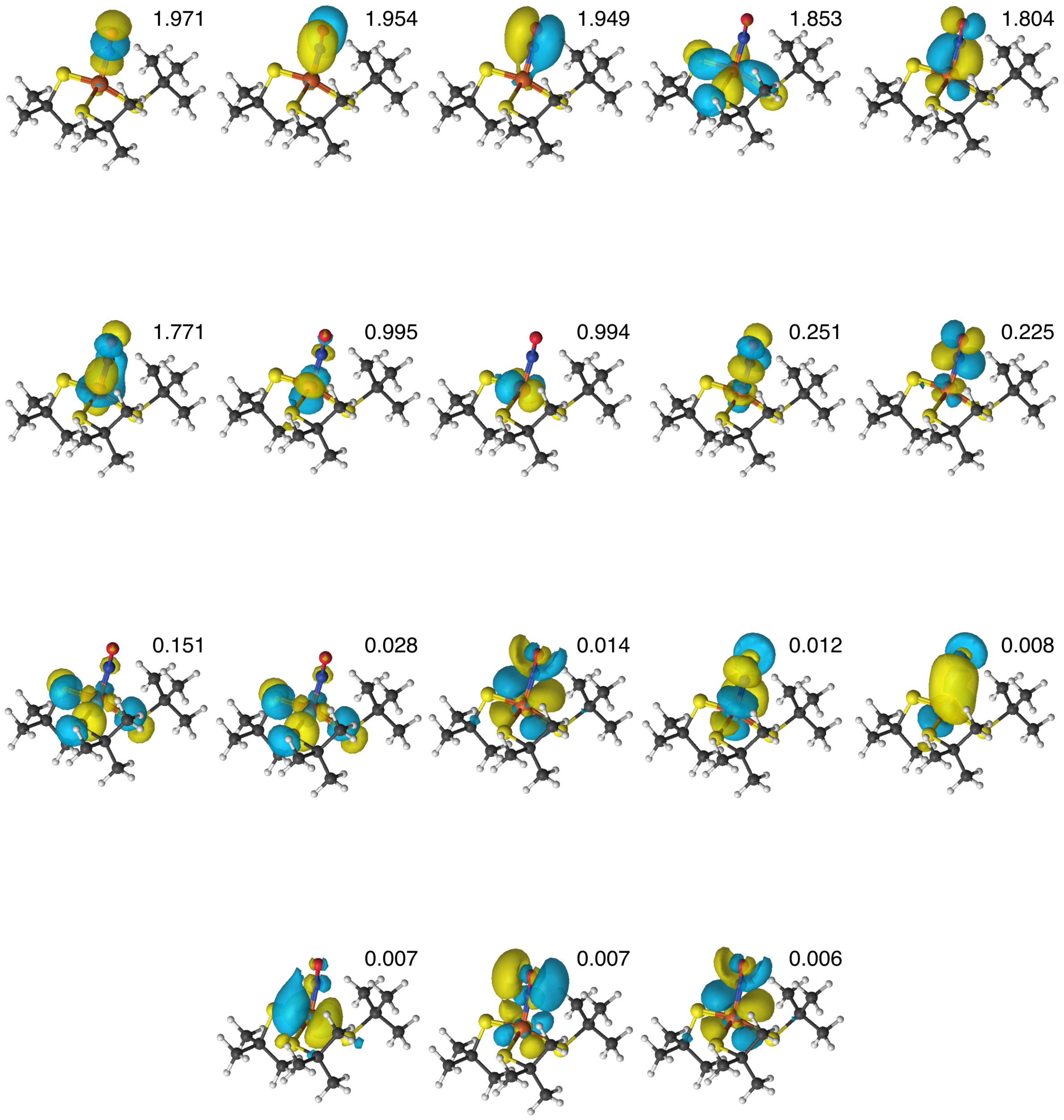

Figure S3: Representations of the 18 active natural orbitals in the DMRG-CASSCF calculations of the $^{3}\{\mathrm{Fe}(\mathrm{S}^{t}\mathrm{Bu})_{3}(\mathrm{NO})\}$ complex (triplet state) with isovalue = 0.04. The number next to each orbital represents the occupation number

Results for $\{Fe(S^tBu)_3(NO)\}^-$

Table S18: Electronic energies E (in E_h) and spin populations for Fe, N, O, and NO for $^4\{Fe(S^tBu)_3(NO)\}^-$ (quartet state), calculated using different DFT functionals and DMRG-CASSCF

Method	Energy (E_h)	Spin Population			
		Fe	N	O	NO
B3LYP	-3061.712071	3.345	-0.573	-0.457	-1.030
B97-D	-3062.454084	2.840	-0.310	-0.254	-0.564
BP86	-3062.755973	2.569	-0.160	-0.153	-0.313
M06-L	-3062.121427	3.165	-0.439	-0.382	-0.821
M06	-3061.696133	3.447	-0.607	-0.498	-1.106
PBE0	-3060.904869	3.549	-0.656	-0.517	-1.173
PBE	-3060.776466	2.569	-0.158	-0.153	-0.311
TPSSh	-3062.429800	3.119	-0.431	-0.339	-0.770
TPSS	-3062.559606	2.641	-0.179	-0.159	-0.338
DMRG-CASSCF	-3054.992587	3.383	-0.295	-0.220	-0.515

Table S19: Electronic energies E (in E_h) and spin populations for Fe, N, O, and NO for $^2\{Fe(S^tBu)_3(NO)\}^-$ (doublet state), calculated using different DFT functionals

Method	Energy (E_h)	Spin Population			
		Fe	N	O	NO
B3LYP	-3061.686723	1.882	-0.491	-0.377	-0.867
B97-D	-3062.437825	1.207	-0.167	-0.132	-0.299
BP86	-3062.750802	1.006	-0.078	-0.072	-0.150
M06-L	-3062.092393	1.445	-0.247	-0.218	-0.464
M06	-3061.654415	2.054	-0.550	-0.442	-0.992
PBE0	-3060.873298	2.184	-0.614	-0.464	-1.078
PBE	-3060.770908	1.002	-0.077	-0.071	-0.148
TPSSh	-3062.413109	1.470	-0.277	-0.213	-0.490
TPSS	-3062.552364	1.044	-0.081	-0.073	-0.154

Table S20: Energy difference between low-spin (doublet) and high-spin (quartet) states of $\{Fe(S^tBu)_3(NO)\}^-$, calculated using different DFT functionals

Functional	ΔE(LS-HS)
B3LYP	15.9
B97-D	10.2
BP86	3.2
M06-L	18.2
M06	26.2
PBE0	19.8
PBE0	3.5
TPSSh	10.5
TPSS	4.5

Table S21: Geometric data for $^4\{Fe(S^tBu)_3(NO)\}^-$, calculated with BP86

d(Fe-N) (Å)	d(N-O) (Å)	ν(N-O) (cm^{-1})
1.676	1.192	1688.05

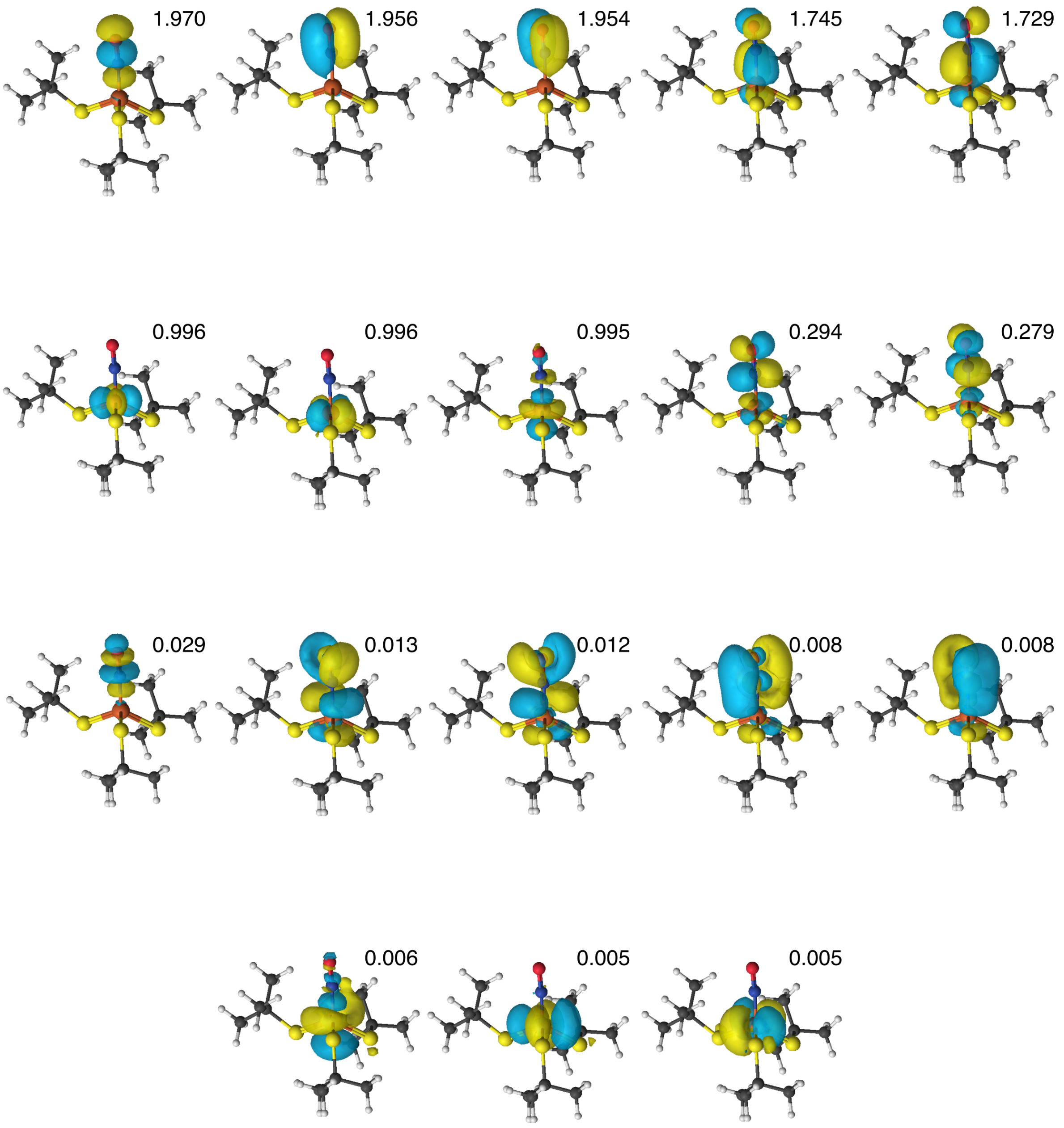

Figure S4: Representations of the 18 active natural orbitals in the DMRG-CASSCF calculations of the $^4\{Fe(S^tBu)_3(NO)\}^-$ complex (quartet state) with isovalue = 0.04. The number next to each orbital represents the occupation number

Results for {Fe[NS3](NO)}

Table S22: Electronic energies E (in E_h) and spin populations for Fe, N, O, and NO for ^{3}{Fe[NS3](NO)} (triplet state), calculated using different DFT functionals and DMRG-CASSCF

Method	Energy (E_h)	Spin Population			
		Fe	N	O	NO
B3LYP	-3453.848786	2.284	-0.339	-0.313	-0.653
B97-D	-3454.493639	1.859	-0.207	-0.171	-0.378
BP86	-3455.152400	1.605	-0.112	-0.098	-0.210
M06-L	-3454.382947	2.160	-0.296	-0.278	-0.574
M06	-3453.701347	2.481	-0.412	-0.384	-0.796
PBE0	-3452.792694	2.548	-0.417	-0.391	-0.808
PBE	-3452.708172	1.606	-0.111	-0.098	-0.209
TPSSh	-3454.802368	2.020	-0.233	-0.206	-0.440
TPSS	-3454.998129	1.655	-0.114	-0.101	-0.214
DMRG-CASSCF	-3445.057181	1.905	-0.155	-0.106	-0.260

Table S23: Electronic energies E (in E_h) and spin populations for Fe, N, O, and NO for ^{1}{Fe[NS3](NO)} (singlet state), calculated using different DFT functionals

Method	Energy (E_h)	Spin Population			
		Fe	N	O	NO
B3LYP	-3453.832121	0	0	0	0
B97-D	-3454.479259	0	0	0	0
BP86	-3455.144632	0	0	0	0
M06-L	-3454.365646	0	0	0	0
M06	-3453.666610	0	0	0	0
PBE0	-3452.771552	0	0	0	0
PBE	-3452.700207	0	0	0	0
TPSSh	-3454.792865	0	0	0	0
TPSS	-3454.992113	0	0	0	0

Table S24: Energy difference between low-spin (singlet) and high-spin (triplet) states of {Fe[NS3](NO)}, calculated using different DFT functionals

Functional	ΔE(LS-HS)
B3LYP	10.5
B97-D	9.0
BP86	4.9
M06-L	10.9
M06	21.8
PBE0	13.3
PBE0	5.0
TPSSh	6.0
TPSS	3.8

Table S25: Geometric data for ^{3}{Fe[NS3](NO)}, calculated using BP86

d(Fe-N) (Å)	d(N-O) (Å)	ν(N-O) (cm^{-1})
1.645	1.158	1864.01

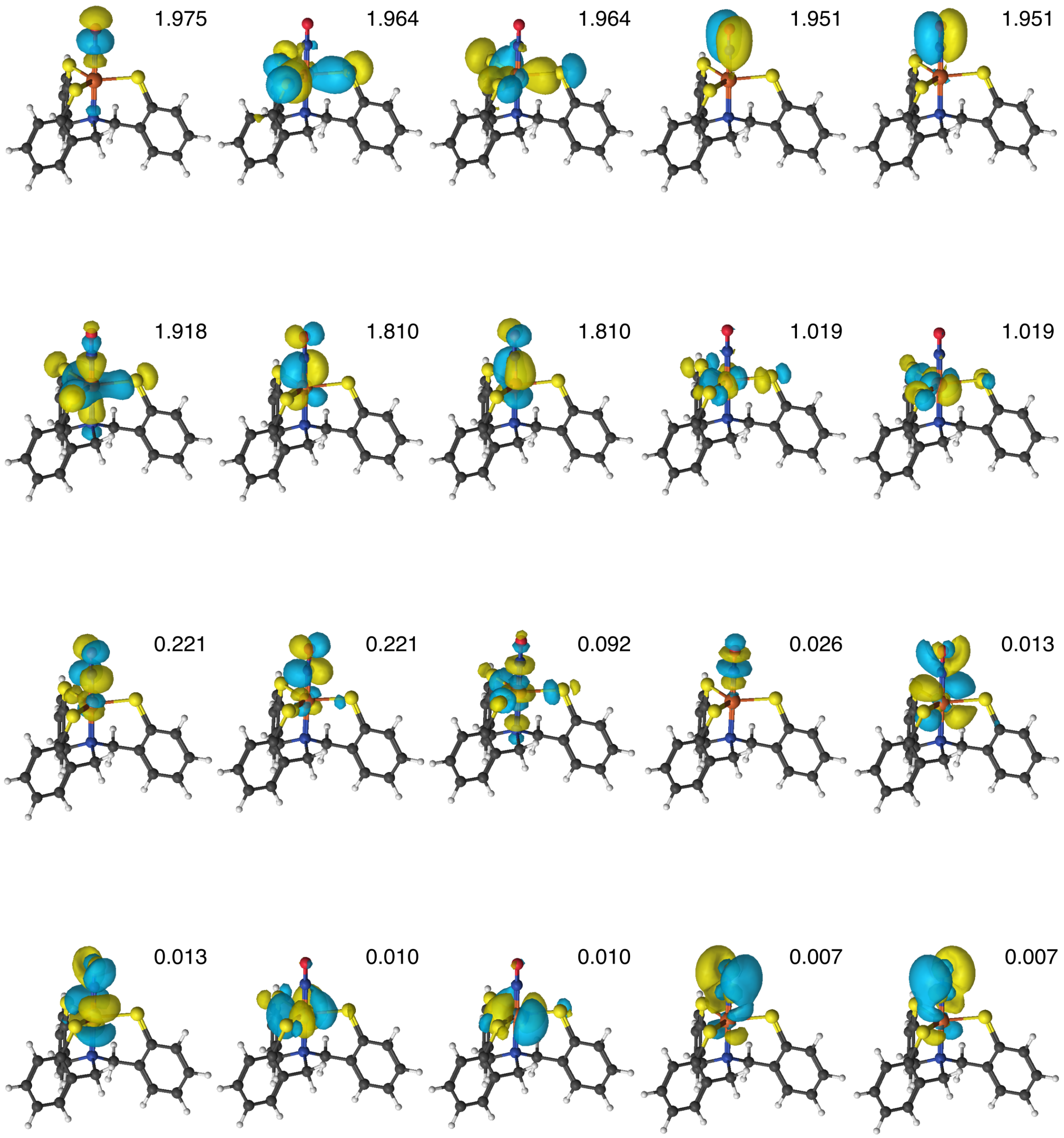

Figure S5: Representations of the 20 active natural orbitals in the DMRG-CASSCF calculations of the $^3\{Fe[NS3](NO)\}$ complex (triplet state) with isovalue = 0.04. The number next to each orbital represents the occupation number

Results for $\{Fe[NS3](NO)\}^-$

Table S26: Electronic energies E (in E_h) and spin populations for Fe, N, O, and NO for $^4\{Fe[NS3](NO)\}^-$ (quartet state), calculated using different DFT functionals and DMRG-CASSCF

Method	Energy (E_h)	Spin Population			
		Fe	N	O	NO
B3LYP	-3453.954658	3.334	-0.448	-0.412	-0.860
B97-D	-3454.584228	2.820	-0.209	-0.195	-0.404
BP86	-3455.240038	2.514	-0.034	-0.069	-0.104
M06-L	-3454.484056	3.151	-0.353	-0.344	-0.697
M06	-3453.820510	3.472	-0.536	-0.478	-1.014
PBE0	-3452.907125	3.569	-0.555	-0.502	-1.057
PBE	-3452.792704	2.519	-0.037	-0.071	-0.109
TPSSh	-3454.896367	2.919	-0.178	-0.193	-0.371
TPSS	-3455.081836	2.548	-0.025	-0.062	-0.086
DMRG-CASSCF	-3445.149133	3.305	-0.245	-0.179	-0.425

Table S27: Electronic energies E (in E_h) and spin populations for Fe, N, O, and NO for $^2\{Fe[NS3](NO)\}^-$ (doublet state), calculated using different DFT functionals

Method	Energy (E_h)	Spin Population			
		Fe	N	O	NO
B3LYP	-3453.927977	1.827	-0.488	-0.364	-0.852
B97-D	-3454.561822	1.217	-0.176	-0.125	-0.301
BP86	-3455.229992	1.022	-0.079	-0.063	-0.142
M06-L	-3454.451474	1.445	-0.253	-0.207	-0.460
M06	-3453.776795	1.846	-0.495	-0.381	-0.876
PBE0	-3452.876205	2.087	-0.605	-0.453	-1.058
PBE	-3452.782038	1.017	-0.078	-0.062	-0.140
TPSSh	-3454.879390	1.478	-0.283	-0.210	-0.493
TPSS	-3455.071959	1.060	-0.076	-0.063	-0.140

Table S28: Energy difference between low-spin (doublet) and high-spin (quartet) states of $\{Fe[NS3](NO)\}^-$, calculated using different DFT functionals

Functional	ΔE(LS-HS)
B3LYP	16.7
B97-D	14.1
BP86	6.3
M06-L	20.4
M06	27.4
PBE0	19.4
PBE0	6.7
TPSSh	10.7
TPSS	6.2

Table S29: Geometric data for $^4\{Fe[NS3](NO)\}^-$, calculated with BP86

d(Fe-N) (Å)	d(N-O) (Å)	ν(N-O) (cm^{-1})
1.723	1.187	1658.49

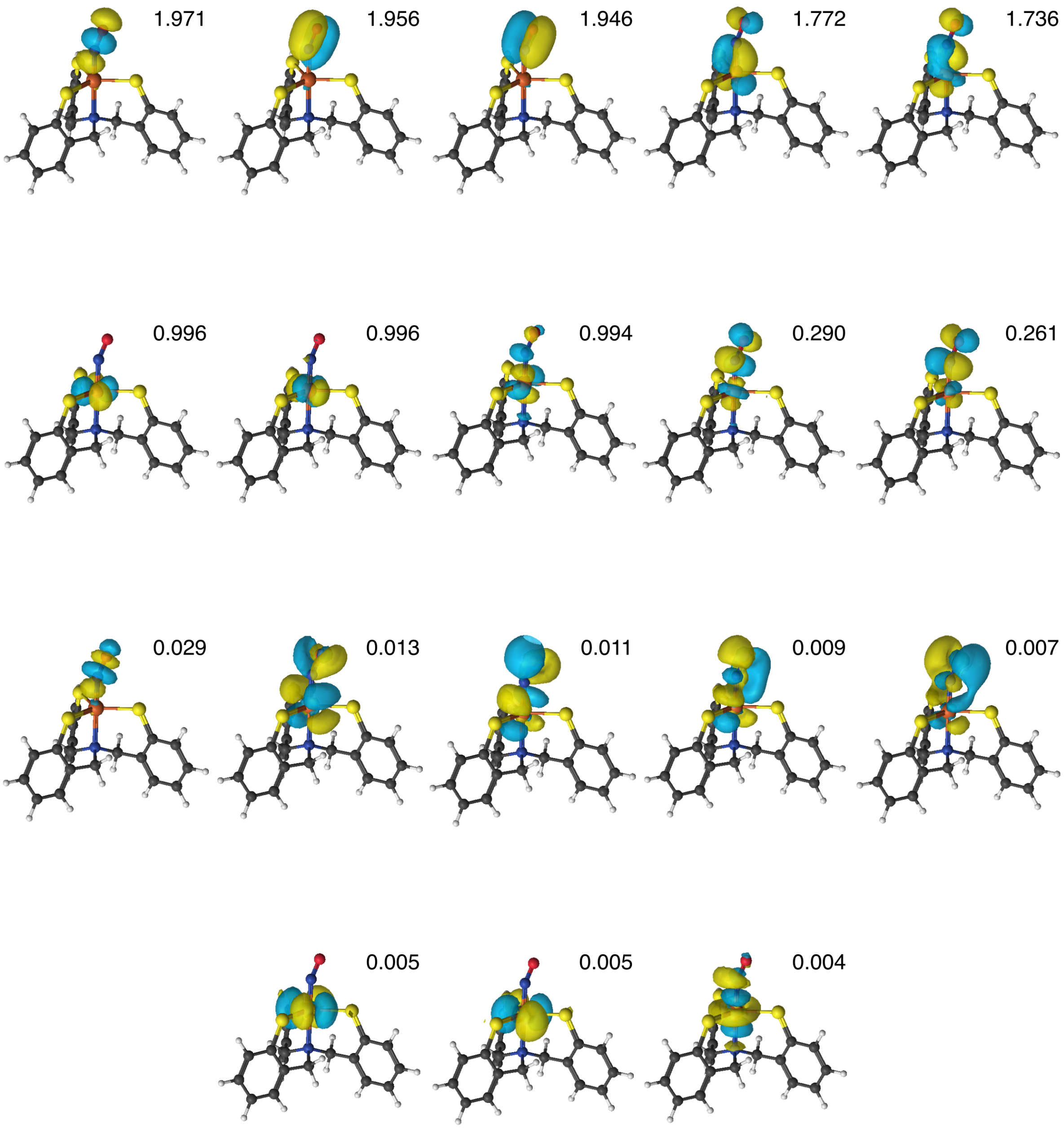

Figure S6: Representations of the 18 active natural orbitals in the DMRG-CASSCF calculations of the $^4\{Fe[NS3](NO)\}^-$ complex (quartet state) with isovalue = 0.04. The number next to each orbital represents the occupation number

Results for {Fe[PS3](NO)}

Table S30: Electronic energies E (in E_h) and spin populations for Fe, N, O, and NO for ^{3}{Fe[PS3](NO)} (triplet state), calculated using different DFT functionals and DMRG-CASSCF

Method	Energy (E_h)	Spin Population			
		Fe	N	O	NO
B3LYP	-3622.560957	2.345	-0.387	-0.341	-0.728
B97-D	-3623.260337	1.754	-0.175	-0.141	-0.316
BP86	-3623.836785	1.512	-0.085	-0.070	-0.154
M06-L	-3623.053310	2.158	-0.312	-0.273	-0.584
M06	-3622.462068	2.510	-0.433	-0.395	-0.828
PBE0	-3621.522361	2.635	-0.470	-0.425	-0.895
PBE	-3621.433279	1.510	-0.082	-0.069	-0.151
TPSSh	-3623.454063	2.020	-0.252	-0.209	-0.460
TPSS	-3623.639860	1.574	-0.090	-0.074	-0.164
DMRG-CASSCF	-3614.247335	2.010	-0.152	-0.112	-0.264

Table S31: Electronic energies E (in E_h) and spin populations for Fe, N, O, and NO for ^{1}{Fe[PS3](NO)} (singlet state), calculated using different DFT functionals

Method	Energy (E_h)	Spin Population			
		Fe	N	O	NO
B3LYP	-3622.545663	0	0	0	0
B97-D	-3623.247681	0	0	0	0
BP86	-3623.830189	0	0	0	0
M06-L	-3623.037356	0	0	0	0
M06	-3622.426242	0	0	0	0
PBE0	-3621.501856	0	0	0	0
PBE	-3621.426582	0	0	0	0
TPSSh	-3623.446311	0	0	0	0
TPSS	-3623.635182	0	0	0	0

Table S32: Energy difference between low-spin (singlet) and high-spin (triplet) states of {Fe[PS3](NO)}, calculated using different DFT functionals

Functional	ΔE(LS-HS)
B3LYP	9.6
B97-D	7.9
BP86	4.1
M06-L	10
M06	22.5
PBE0	12.9
PBE0	4.2
TPSSh	4.9
TPSS	2.9

Table S33: Geometric data for ^{3}{Fe[PS3](NO)}, calculated with BP86

d(Fe-N) (Å)	d(N-O) (Å)	ν(N-O) (cm^{-1})
1.670	1.161	1847.61

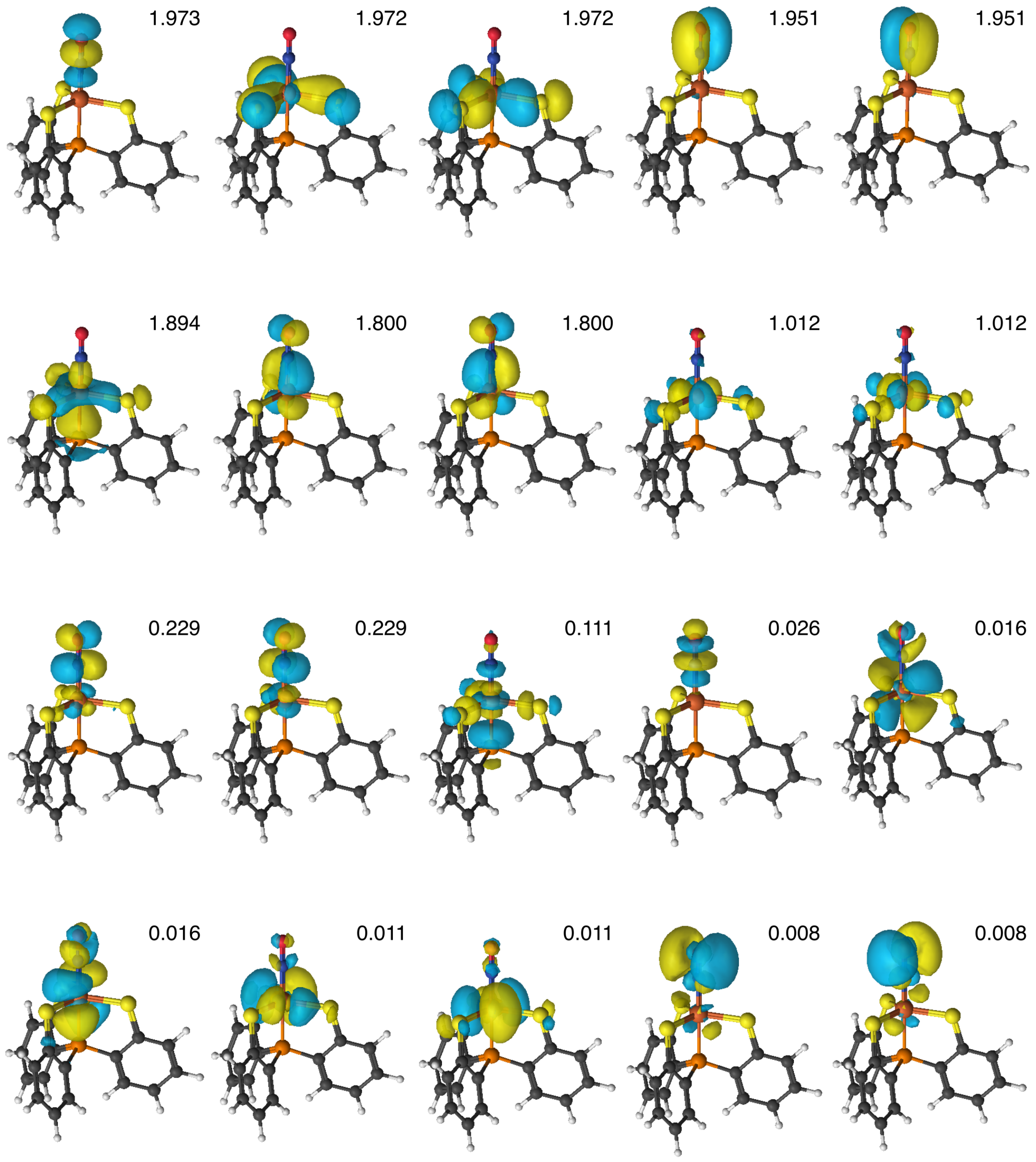

Figure S7: Representations of the 20 active natural orbitals in the DMRG-CASSCF calculations of the ^{3}\{Fe[PS3](NO)\} complex (triplet state) with isovalue = 0.04. The number next to each orbital represents the occupation number

Results for $\{Fe[PS3](NO)\}^{-}$

Table S34: Electronic energies E (in E_h) and spin populations for Fe, N, O, and NO for $^4\{Fe[PS3](NO)\}^{-}$ (quartet state), calculated using different DFT functionals

Method	Energy (E_h)	Spin Population			
		Fe	N	O	NO
B3LYP	-3622.645041	2.300	0.308	0.124	0.432
B97-D	-3623.329685	2.311	0.133	0.045	0.178
BP86	-3623.907160	2.085	0.228	0.121	0.349
M06-L	-3623.126645	2.574	0.040	-0.038	0.002
M06	-3622.543567	2.640	0.095	-0.020	0.075
PBE0	-3621.607885	2.439	0.292	0.097	0.389
PBE	-3621.499780	2.095	0.220	0.116	0.336
TPSSh	-3623.530697	2.260	0.287	0.134	0.422
TPSS	-3623.707447	2.112	0.257	0.137	0.394

Table S35: Electronic energies E (in E_h) and spin populations for Fe, N, O, and NO for $^2\{Fe[PS3](NO)\}^{-}$ (doublet state), calculated using different DFT functionals and DMRG-CASSCF

Method	Energy (E_h)	Spin Population			
		Fe	N	O	NO
B3LYP	-3622.645545	1.959	-0.580	-0.400	-0.980
B97-D	-3623.335018	1.350	-0.261	-0.170	-0.430
BP86	-3623.919769	1.100	-0.144	-0.095	-0.240
M06-L	-3623.128693	1.638	-0.376	-0.275	-0.650
M06	-3622.542484	2.018	-0.577	-0.421	-0.998
PBE0	-3621.610122	2.196	-0.673	-0.468	-1.142
PBE	-3621.512311	1.089	-0.139	-0.093	-0.232
TPSSh	-3623.536884	1.684	-0.429	-0.284	-0.713
TPSS	-3623.719146	1.166	-0.160	-0.106	-0.266
DMRG-CASSCF	-3614.293752	1.253	-0.144	-0.088	-0.233

Table S36: Energy difference between low-spin (doublet) and high-spin (quartet) states of $\{Fe[PS3](NO)\}^{-}$, calculated using different DFT functionals

Functional	ΔE(LS-HS)
B3LYP	-0.3
B97-D	-3.3
BP86	-7.9
M06-L	-1.3
M06	0.7
PBE0	-1.4
PBE0	-7.9
TPSSh	-3.9
TPSS	-7.3

Table S37: Geometric data for $^{2}\{Fe[PS3](NO)\}^{-}$, calculated with BP86

d(Fe-N) (Å)	d(N-O) (Å)	ν(N-O) (cm^{-1})
1.66	1.181	1760.71

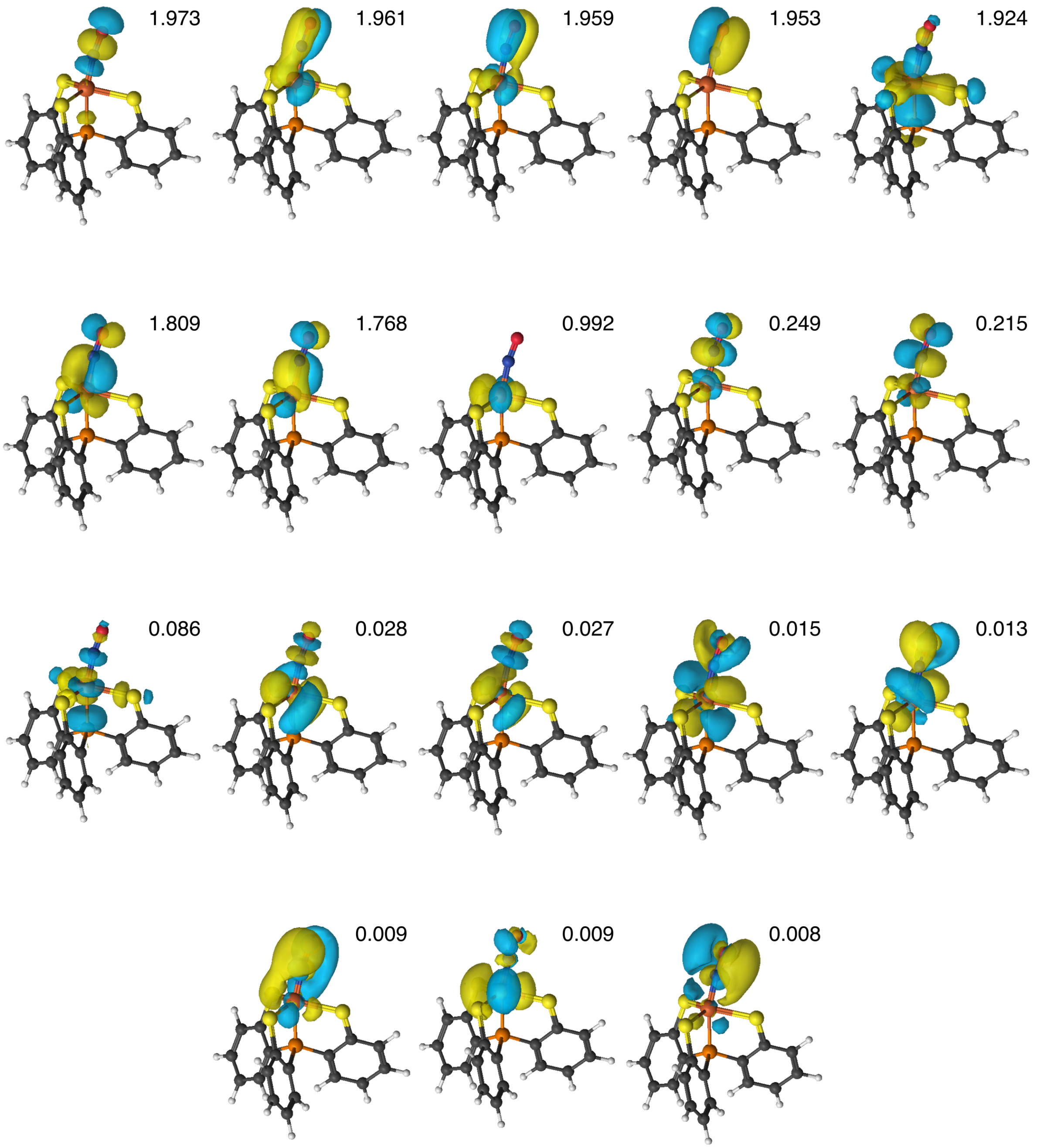

Figure S8: Representations of the 18 active natural orbitals in the DMRG-CASSCF calculations of the $^2\{Fe[PS3](NO)\}^-$ complex (doublet state) with isovalue = 0.04. The number next to each orbital represents the occupation number

Results for $\{Fe(CN)_5(NO)\}^{2-}$

Table S38: Electronic energies E (in E_h) and spin populations for Fe, N, O and, NO for $^3\{Fe(CN)_5(NO)\}^{2-}$ (triplet state), calculated using different DFT functionals

Method	Energy (E_h)	Spin Population			
		Fe	N	O	NO
B3LYP	-1857.836707	1.296	0.493	0.259	0.752
B97-D	-1858.312217	1.422	0.364	0.200	0.564
BP86	-1858.522571	1.305	0.400	0.229	0.628
M06-L	-1858.078009	1.421	0.377	0.209	0.586
M06	-1857.741408	1.362	0.446	0.231	0.677
PBE0	-1857.261858	1.341	0.501	0.256	0.757
PBE	-1857.351775	1.311	0.393	0.226	0.619
TPSSh	-1858.258625	1.296	0.469	0.251	0.720
TPSS	-1858.399058	1.269	0.428	0.239	0.667

Table S39: Electronic energies E (in E_h) and spin populations for Fe, N, O, and NO for $^1\{Fe(CN)_5(NO)\}^{2-}$ (singlet state), calculated using different DFT functionals and DMRG-CASSCF

Method	Energy (E_h)	Spin Population			
		Fe	N	O	NO
B3LYP	-1857.862696	0	0	0	0
B97-D	-1858.358477	0	0	0	0
BP86	-1858.571786	0	0	0	0
M06-L	-1858.117843	0	0	0	0
M06	-1857.774417	0	0	0	0
PBE0	-1857.286059	0	0	0	0
PBE	-1857.401868	0	0	0	0
TPSSh	-1858.294670	0	0	0	0
TPSS	-1858.444126	0	0	0	0
DMRG-CASSCF	-1853.703247	0	0	0	0

Table S40: Energy difference between low-spin (singlet) and high-spin (triplet) states of $\{Fe(CN)_5(NO)\}^{2-}$, calculated using different DFT functionals

Functional	ΔE(LS-HS)
B3LYP	-16.3
B97-D	-29.0
BP86	-30.9
M06-L	-25.0
M06	-20.7
PBE0	-15.2
PBE0	-31.4
TPSSh	-22.6
TPSS	-28.3

Table S41: Geometric data for $^1\{Fe(CN)_5(NO)\}^{2-}$, calculated with BP86

d(Fe-N) (Å)	d(N-O) (Å)	ν(N-O) (cm^{-1})
1.637	1.163	1855.71

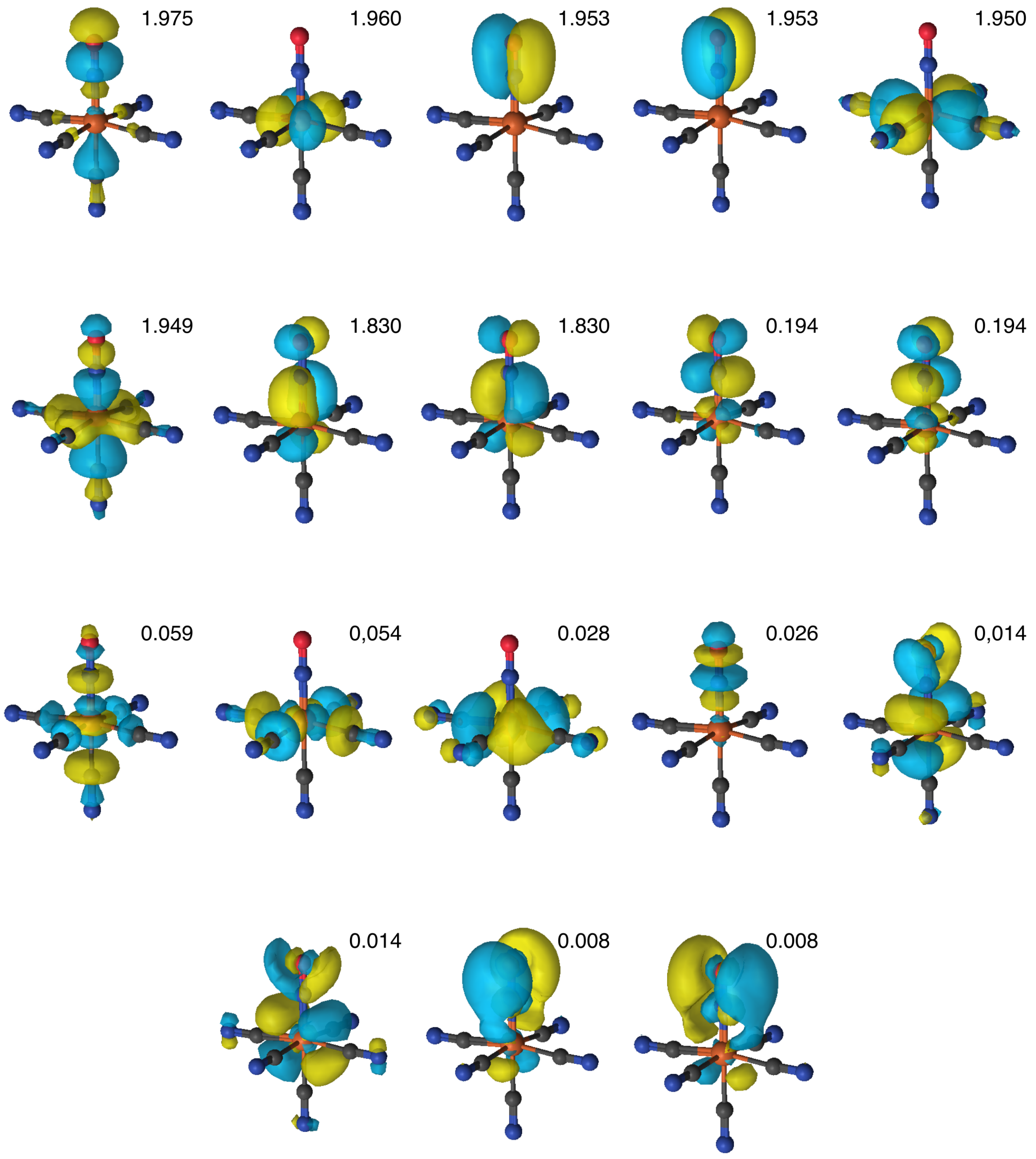

Figure S9: Representations of the 18 active natural orbitals in the DMRG-CASSCF calculations of the $^{1}\{Fe(CN)_5(NO)\}^{2-}$ complex (singlet state) with isovalue = 0.04. The number next to each orbital represents the occupation number

Results for $\{Fe(CN)_4(NO)\}^{2-}$

Table S42: Electronic energies E (in E_h) and spin populations for Fe, N, O, and NO for $^4\{Fe(CN)_4(NO)\}^{2-}$ (quartet state), calculated using different DFT functionals

Method	Energy (E_h)	Spin Population			
		Fe	N	O	NO
B3LYP	-1764.995689	2.334	0.559	0.191	0.750
B97-D	-1765.477976	2.345	0.551	0.194	0.745
BP86	-1765.608995	2.205	0.583	0.229	0.812
M06-L	-1765.195470	2.404	0.501	0.177	0.678
M06	-1764.915000	2.504	0.472	0.137	0.609
PBE0	-1764.485758	2.442	0.537	0.165	0.701
PBE	-1764.551010	2.203	0.579	0.227	0.806
TPSSh	-1765.357951	2.281	0.594	0.219	0.812
TPSS	-1765.476429	2.191	0.603	0.239	0.842

Table S43: Electronic energies E (in E_h) and spin populations for Fe, N, O, and NO for $^2\{Fe(CN)_4(NO)\}^{2-}$ (doublet state), calculated using different DFT functionals and DMRG-CASSCF

Method	Energy (E_h)	Spin Population			
		Fe	N	O	NO
B3LYP	-1765.029385	1.775	-0.410	-0.326	-0.736
B97-D	-1765.532964	1.247	-0.105	-0.121	-0.226
BP86	-1765.669976	1.033	0.000	-0.061	-0.061
M06-L	-1765.241472	1.399	-0.186	-0.168	-0.354
M06	-1764.956924	1.882	-0.487	-0.375	-0.863
PBE0	-1764.519273	2.039	-0.537	-0.405	-0.941
PBE	-1764.612975	1.024	0.000	-0.062	-0.062
TPSSh	-1765.400227	1.302	-0.133	-0.134	-0.267
TPSS	-1765.531297	1.026	0.010	-0.046	-0.036
DMRG-CASSCF	-1761.367641	1.102	-0.057	-0.051	-0.108

Table S44: Energy difference between low-spin (doublet) and high-spin (quartet) states of $\{Fe(CN)_4(NO)\}^{2-}$, calculated using different DFT functionals

Functional	ΔE(LS-HS)
B3LYP	-21.1
B97-D	-34.5
BP86	-38.3
M06-L	-28.9
M06	-26.3
PBE0	-21.0
PBE0	-38.9
TPSSh	-26.5
TPSS	-34.4

Table S45: Geometric data for $^2\{Fe(CN)_4(NO)\}^{2-}$, calculated using BP86

d(Fe-N) (Å)	d(N-O) (Å)	ν(N-O) (cm^{-1})
1.653	1.186	1719.97

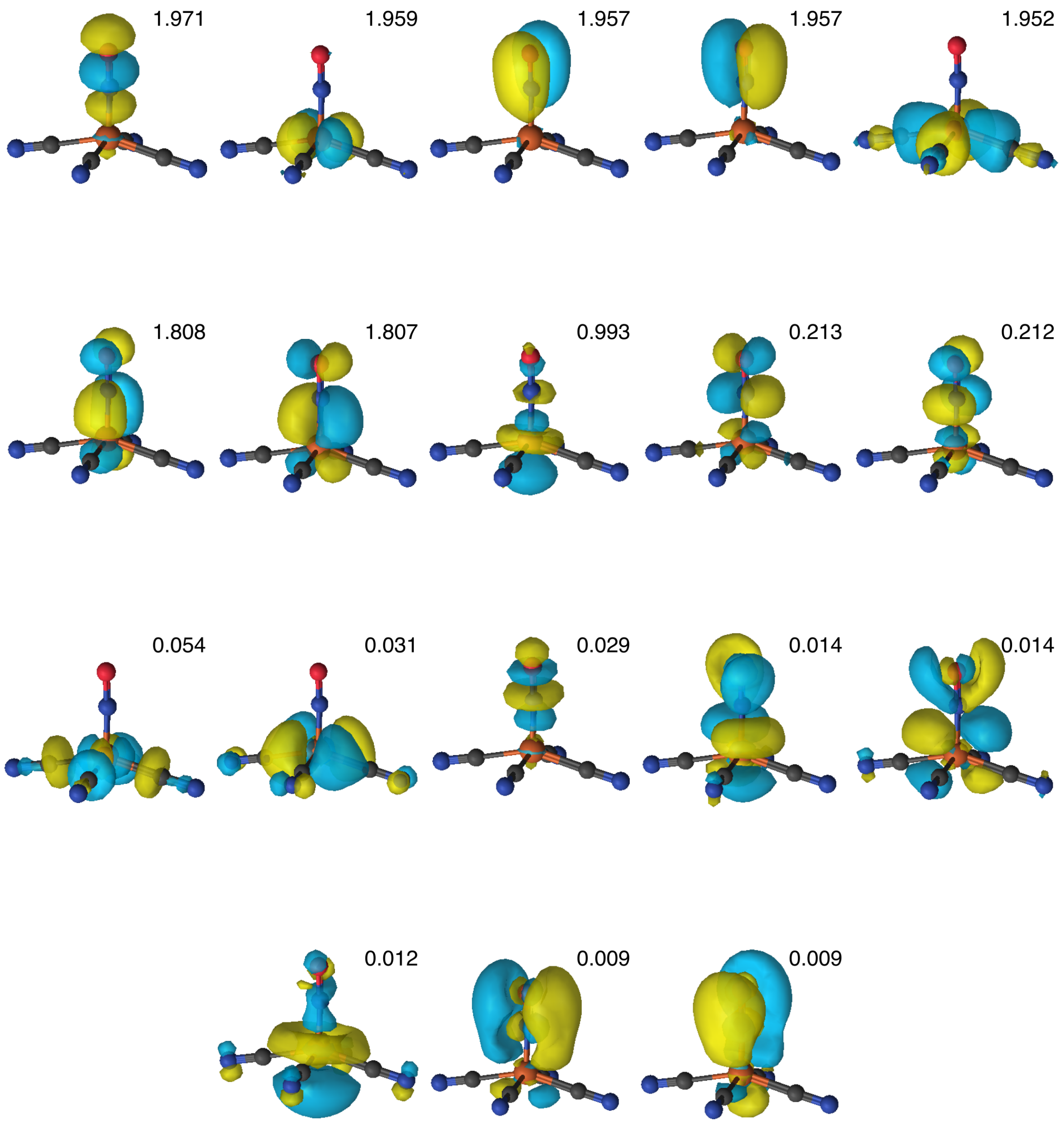

Figure S10: Representations of the 18 active natural orbitals in the DMRG-CASSCF calculations of the $^{2}\{Fe(CN)_4(NO)\}^{2-}$ complex (doublet state) with isovalue = 0.04. The number next to each orbital represents the occupation number

Results for $\{Fe[TIMEN](CH_3CN)(NO)\}^{3+}$

Table S46: Electronic energies E (in E_h) and spin populations for Fe, N, O, and NO for $^3\{Fe[TIMEN](CH_3CN)(NO)\}^{3+}$ (triplet state), calculated using different DFT functionals

Method	Energy (E_h)	Spin Population			
		Fe	N	O	NO
B3LYP	-3539.638729	1.668	0.249	0.165	0.414
B97-D	-3539.973671	1.861	0.048	0.000	0.048
BP86	-3541.506559	1.626	0.132	0.063	0.195
M06-L	-3540.567920	1.989	0.072	-0.024	0.049
M06	-3539.158835	2.601	-0.102	-0.301	-0.402
PBE0	-3538.080359	1.759	0.253	0.166	0.419
PBE	-3537.965707	1.628	0.125	0.060	0.186
TPSSh	-3541.259607	1.683	0.211	0.126	0.337
TPSS	-3541.573334	1.578	0.167	0.089	0.256

Table S47: Electronic energies E (in E_h) and spin populations for Fe, N, O, and NO for $^1\{Fe[TIMEN](CH_3CN)(NO)\}^{3+}$ (singlet state), calculated using different DFT functionals and DMRG-CASSCF

Method	Energy (E_h)	Spin Population			
		Fe	N	O	NO
B3LYP	-3539.649076	0	0	0	0
B97-D	-3539.985612	0	0	0	0
BP86	-3541.525233	0	0	0	0
M06-L	-3540.585238	0	0	0	0
M06	-3539.174381	0	0	0	0
PBE0	-3538.093855	0	0	0	0
PBE	-3537.989328	0	0	0	0
TPSSh	-3541.278487	0	0	0	0
TPSS	-3541.595043	0	0	0	0
DMRG-CASSCF	-3525.039363	0	0	0	0

Table S48: Energy difference between low-spin (singlet) and high-spin (triplet) states of $\{Fe[TIMEN](CH_3CN)(NO)\}^{3+}$, calculated using different DFT functionals

Functional	ΔE(LS-HS)
B3LYP	-6.5
B97-D	-7.5
BP86	-11.7
M06-L	-10.9
M06	-9.8
PBE0	-8.5
PBE0	-14.8
TPSSh	-11.8
TPSS	-13.6

Table S49: Geometric data for $^1\{Fe[TIMEN](CH_3CN)(NO)\}^{3+}$, calculated with BP86

d(Fe-N) (Å)	d(N-O) (Å)	ν(N-O) (cm^{-1})
1.636	1.140	1946.95

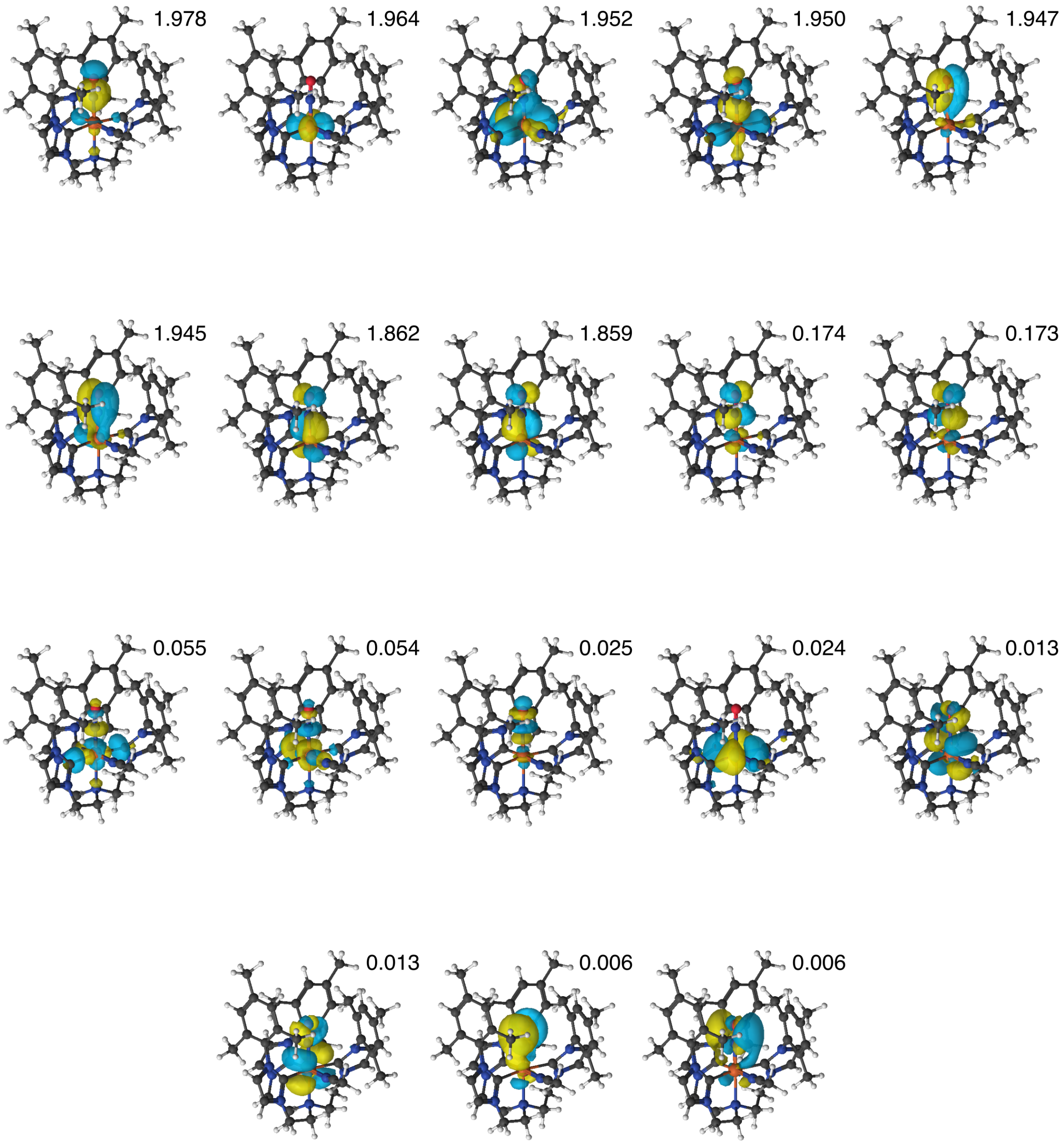

Figure S11: Representations of the 18 active natural orbitals in the DMRG-CASSCF calculations of the $^{1}\{Fe[TIMEN](CH_3CN)(NO)\}^{3+}$ complex (singlet state) with isovalue = 0.04. The number next to each orbital represents the occupation number

Results for $\{Fe[TIMEN](NO)\}^{2+}$

Table S50: Electronic energies E (in E_h) and spin populations for Fe, N, O, and NO for $^4\{Fe[TIMEN](NO)\}^{2+}$ (quartet state), calculated using different DFT functionals and DMRG-CASSCF

Method	Energy (E_h)	Spin Population			
		Fe	N	O	NO
B3LYP	-3407.320005	3.566	-0.400	-0.417	-0.817
B97-D	-3407.657597	3.206	-0.257	-0.236	-0.493
BP86	-3409.078907	2.970	-0.149	-0.141	-0.290
M06-L	-3408.187281	3.502	-0.320	-0.372	-0.692
M06	-3406.885061	3.762	-0.430	-0.496	-0.926
PBE0	-3405.860199	3.779	-0.451	-0.487	-0.938
PBE	-3405.718779	2.953	-0.147	-0.140	-0.287
TPSSh	-3408.84233	3.416	-0.309	-0.305	-0.614
TPSS	-3409.127189	3.019	-0.158	-0.147	-0.305
DMRG-CASSCF	-3393.494587	3.306	-0.220	-0.197	-0.417

Table S51: Electronic energies E (in E_h) and spin populations for Fe, N, O, and NO for $^2\{Fe[TIMEN](NO)\}^{2+}$ (doublet state), calculated using different DFT functionals

Method	Energy (E_h)	Spin Population			
		Fe	N	O	NO
B3LYP	-3407.297604	0.985	0.059	0.000	0.059
B97-D	-3407.644843	1.005	0.000	-0.014	-0.014
BP86	-3409.082942	0.933	0.039	0.012	0.051
M06-L	-3408.166023	1.113	-0.012	-0.022	-0.034
M06	-3406.845531	1.331	-0.184	-0.187	-0.371
PBE0	-3405.830884	1.062	0.033	-0.017	0.016
PBE	-3405.721290	0.924	0.037	0.011	0.048
TPSSh	-3408.836403	0.976	0.057	0.016	0.073
TPSS	-3409.131721	0.928	0.054	0.021	0.075

Table S52: Energy difference between low-spin (doublet) and high-spin (quartet) states of $\{Fe[TIMEN](NO)\}^{2+}$, calculated using different DFT functionals

Functional	ΔE(LS-HS)
B3LYP	14.1
B97-D	8.0
BP86	-2.5
M06-L	13.3
M06	24.8
PBE0	18.4
PBE0	-1.6
TPSSh	3.7
TPSS	-2.8

Table S53: Geometric data for $^4\{Fe[TIMEN](NO)\}^{2+}$, calculated with BP86

d(Fe-N) (Å)	d(N-O) (Å)	ν(N-O) (cm^{-1})
1.699	1.158	1829.29

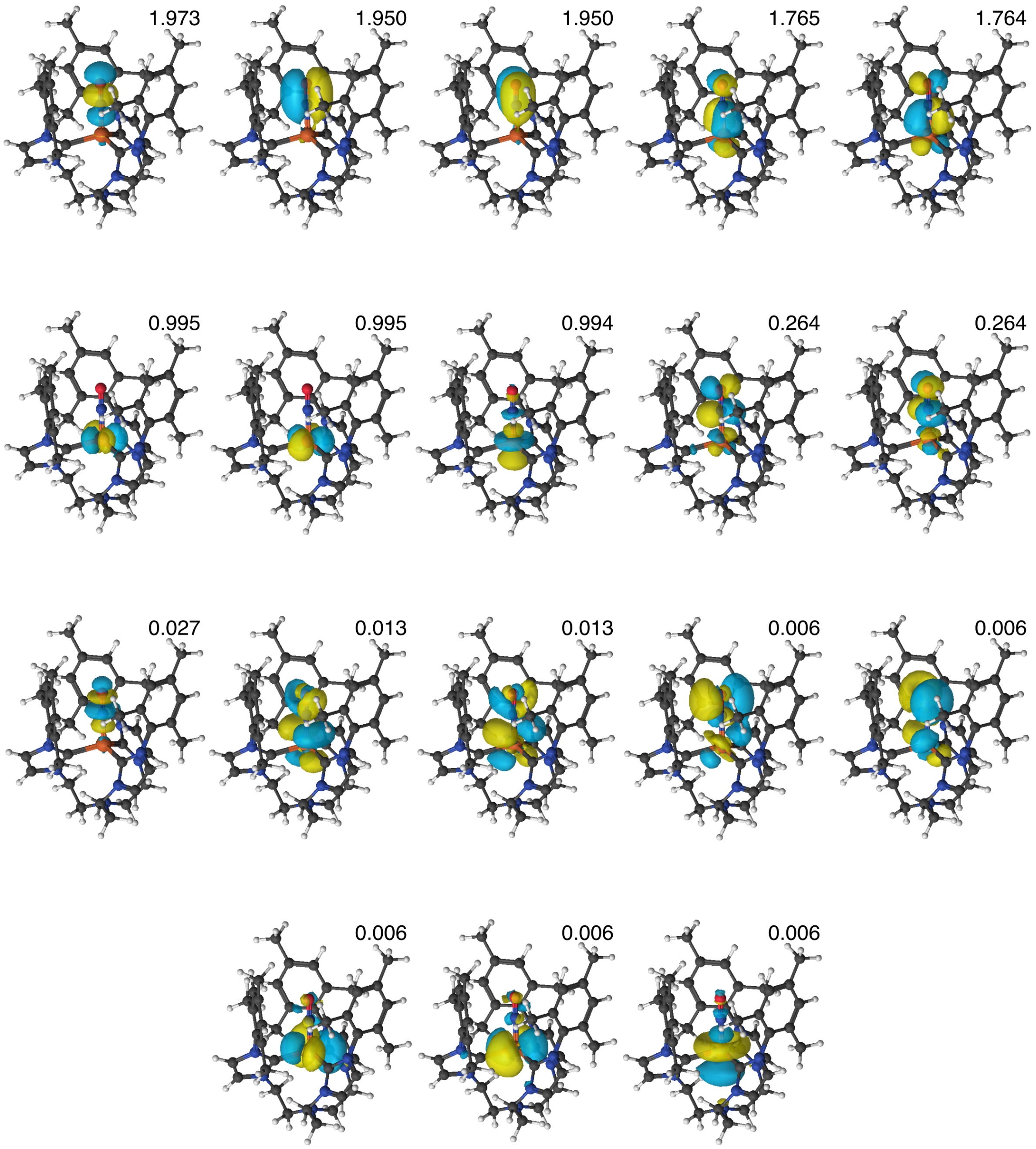

Figure S12: Representations of the 18 active natural orbitals in the DMRG-CASSCF calculations of the $^4\{Fe[TIMEN](NO)\}^{2+}$ complex (quartet state) with isovalue = 0.04. The number next to each orbital represents the occupation number

Results for $\{Fe[TIMEN](NO)\}^+$

Table S54: Electronic energies E (in E_h) and spin populations for Fe, N, O, and NO for $^3\{Fe[TIMEN](NO)\}^+$ (triplet state), calculated using different DFT functionals and DMRG-CASSCF

Method	Energy (E_h)	Spin Population			
		Fe	N	O	NO
B3LYP	-3407.588242	2.791	-0.443	-0.380	-0.823
B97-D	-3407.929422	2.335	-0.178	-0.166	-0.344
BP86	-3409.363601	2.083	-0.058	-0.074	-0.132
M06-L	-3408.453937	2.645	-0.256	-0.281	-0.537
M06	-3407.153355	3.005	-0.500	-0.493	-0.993
PBE0	-3406.127116	3.081	-0.572	-0.486	-1.059
PBE	-3405.998230	2.065	-0.057	-0.074	-0.131
TPSSh	-3409.115433	2.475	-0.251	-0.221	-0.472
TPSS	-3409.405443	2.093	-0.061	-0.072	-0.133
DMRG-CASSCF	-3393.721862	2.245	-0.174	-0.136	-0.310

Table S55: Electronic energies E (in E_h) and spin populations for Fe, N, O, and NO for $^1\{Fe[TIMEN](NO)\}^+$ (singlet state), calculated using different DFT functionals

Method	Energy (E_h)	Spin Population			
		Fe	N	O	NO
B3LYP	-3407.564762	0	0	0	0
B97-D	-3407.921413	0	0	0	0
BP86	-3409.361927	0	0	0	0
M06-L	-3408.432602	0	0	0	0
M06	-3407.117182	0	0	0	0
PBE0	-3406.093182	0	0	0	0
PBE	-3405.995940	0	0	0	0
TPSSh	-3409.100003	0	0	0	0
TPSS	-3409.399381	0	0	0	0

Table S56: Energy difference between low-spin (singlet) and high-spin (triplet) states of $\{Fe[TIMEN](NO)\}^{+}$, calculated using different DFT functionals

Functional	ΔE(LS-HS)
B3LYP	14.7
B97-D	5.0
BP86	1.1
M06-L	13.4
M06	22.7
PBE0	21.3
PBE0	1.4
TPSSh	9.7
TPSS	3.8

Table S57: Geometric data for $^{3}\{Fe[TIMEN](NO)\}^{+}$, calculated using BP86

d(Fe-N) (Å)	d(N-O) (Å)	ν(N-O) (cm^{-1})
1.665	1.181	1732.13

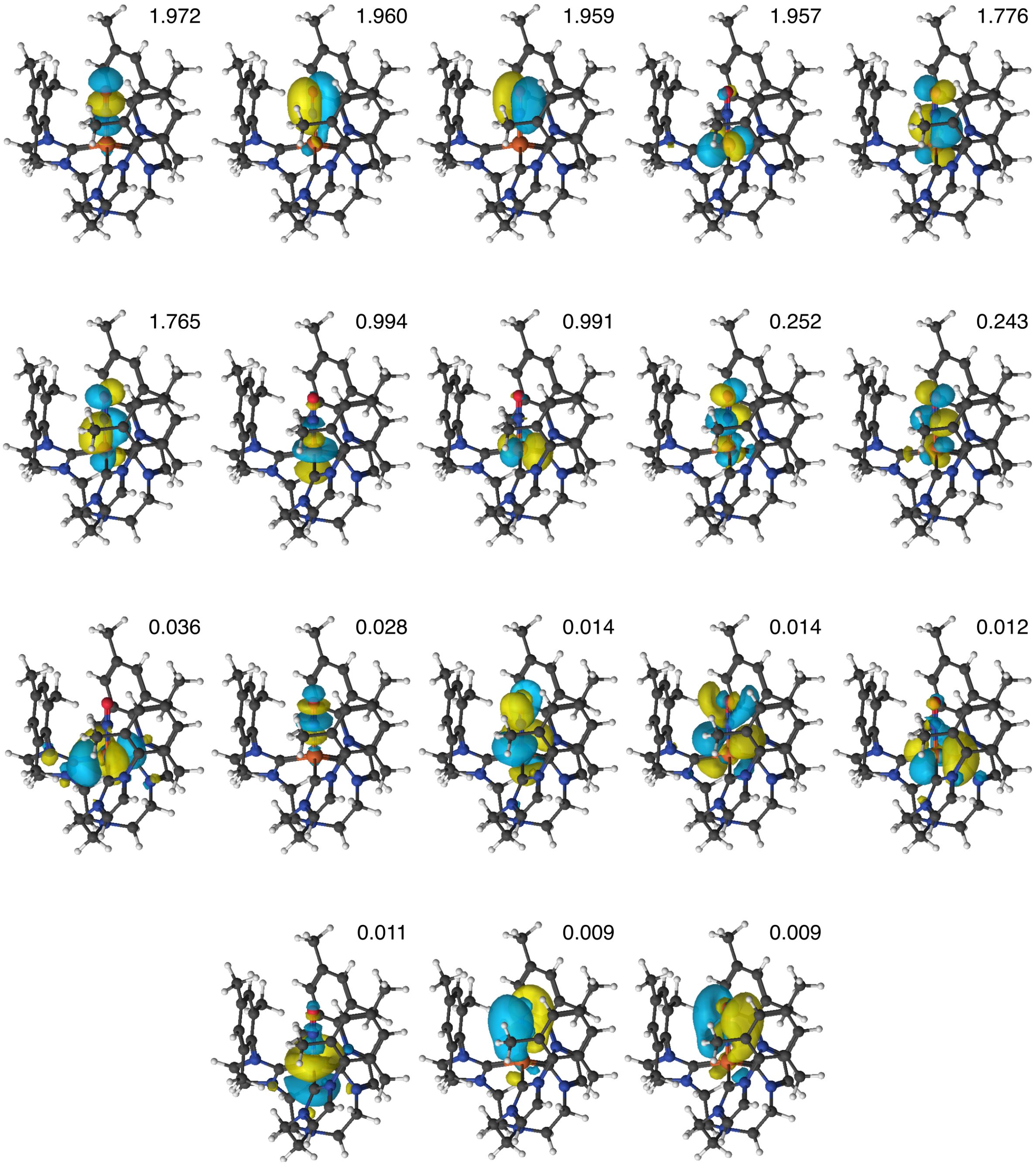

Figure S13: Representations of the 18 active natural orbitals in the DMRG-CASSCF calculations of the $^3\{Fe[TIMEN](NO)\}^+$ complex (triplet state) with isovalue = 0.04. The number next to each orbital represents the occupation number

Results for {Fe[TIMEN](NO)}

Table S58: Electronic energies E (in E_h) and spin populations for Fe, N, O, and NO for ^{2}{Fe[TIMEN](NO)} (doublet state), calculated using different DFT functionals and DMRG-CASSCF

Method	Energy (E_h)	Spin Population			
		Fe	N	O	NO
B3LYP	-3407.705317	1.976	-0.459	-0.320	-0.779
B97-D	-3408.062205	1.429	-0.113	-0.112	-0.225
BP86	-3409.508400	1.206	-0.028	-0.054	-0.081
M06-L	-3408.575594	1.783	-0.230	-0.216	-0.445
M06	-3407.272438	2.283	-0.436	-0.442	-0.878
PBE0	-3406.240945	2.317	-0.609	-0.414	-1.023
PBE	-3406.138790	1.193	-0.028	-0.053	-0.081
TPSSh	-3409.241067	1.564	-0.219	-0.167	-0.386
TPSS	-3409.540223	1.211	-0.033	-0.052	-0.085
DMRG-CASSCF	-3393.784293	1.146	-0.073	-0.055	-0.128

Table S59: Geometric data for ^{2}{Fe[TIMEN](NO)}, calculated with BP86

d(Fe-N) (Å)	d(N-O) (Å)	ν(N-O) (cm^{-1})
1.655	1.181	1633.38

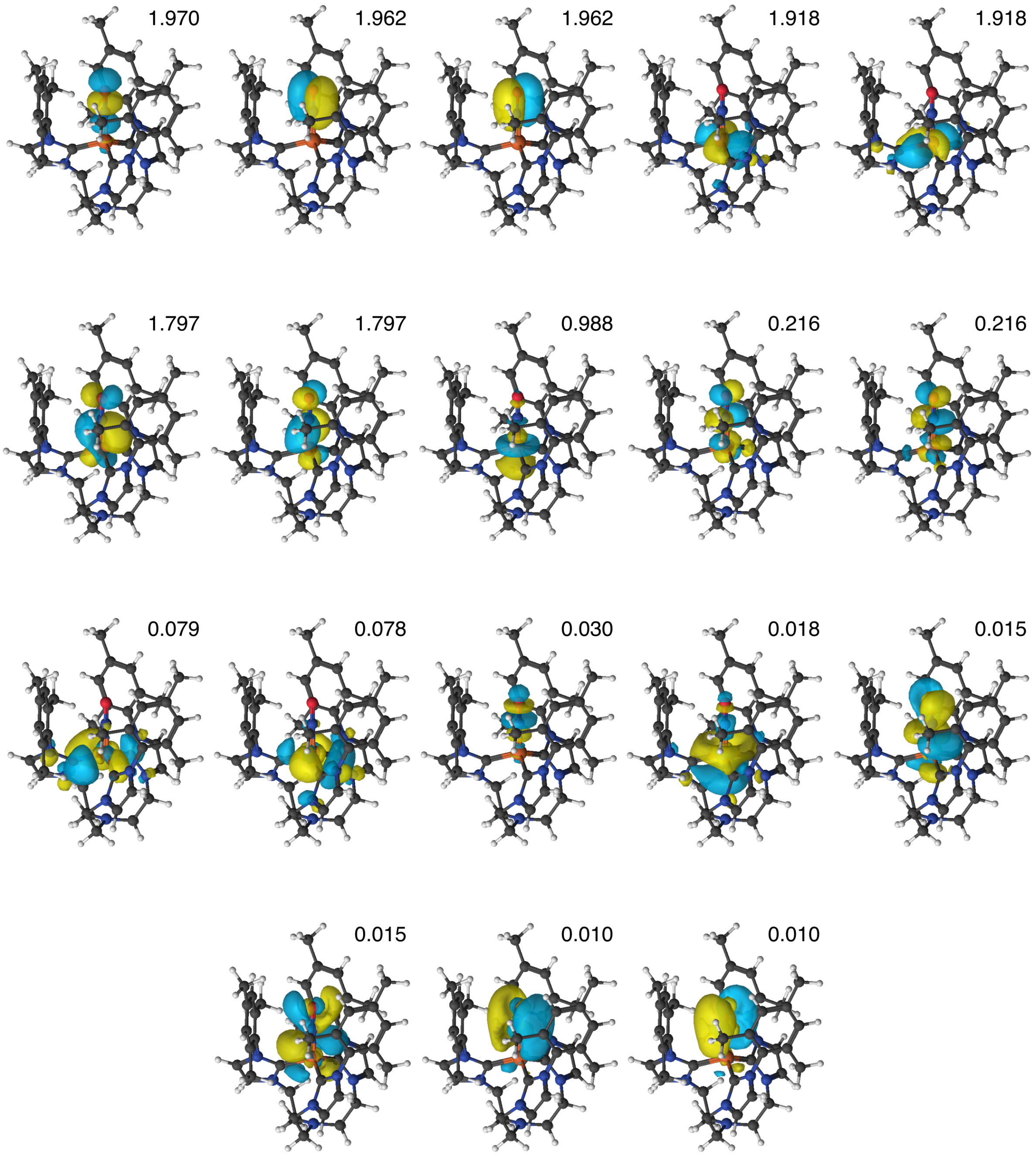

Figure S14: Representations of the 18 active natural orbitals in the DMRG-CASSCF calculations of the ${}^{2}\{Fe[TIMEN](NO)\}$ complex (doublet state) with isovalue = 0.04. The number next to each orbital represents the occupation number

Results for $\{Fe[TIMEN](NO)\}^-$

Table S60: Electronic energies E (in E_h) and spin populations for Fe, N, O, and NO for $^1\{Fe[TIMEN](NO)\}^-$ (singlet state), calculated using different DFT functionals and DMRG-CASSCF

Method	Energy (E_h)	Spin Population			
		Fe	N	O	NO
B3LYP	-3407.698336	0	0	0	0
B97-D	-3408.073856	0	0	0	0
BP86	-3409.532044	0	0	0	0
M06-L	-3408.576474	0	0	0	0
M06	-3407.262484	0	0	0	0
PBE0	-3406.228527	0	0	0	0
PBE	-3406.157149	0	0	0	0
TPSSh	-3409.245499	0	0	0	0
TPSS	-3409.554201	0	0	0	0
DMRG-CASSCF	-3393.729606	0	0	0	0

Table S61: Geometric data for $^1\{Fe[TIMEN](NO)\}^-$, calculated with BP86

d(Fe-N) (Å)	d(N-O) (Å)	ν(N-O) (cm^{-1})
1.652	1.225	1534.62

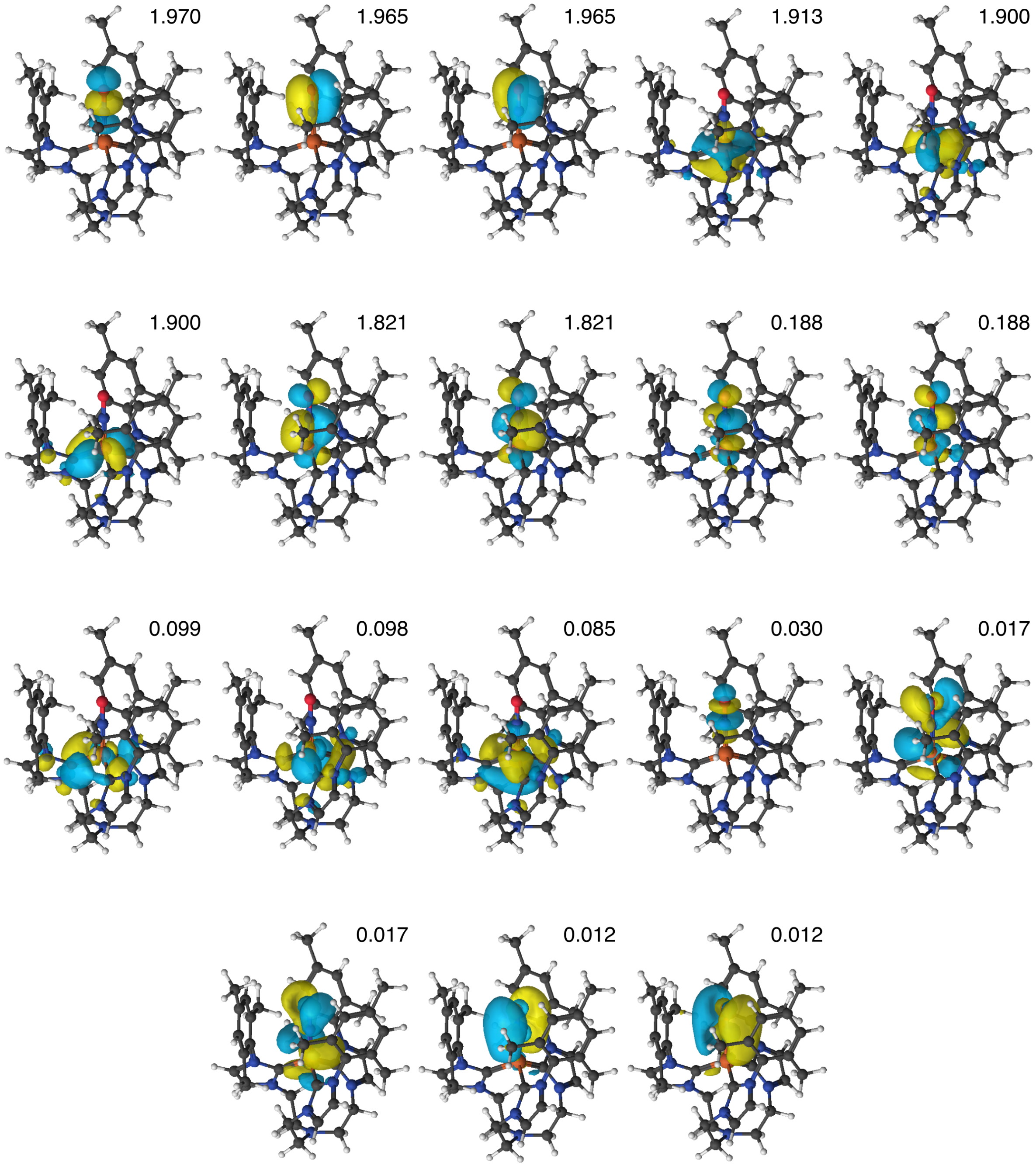

Figure S15: Representations of the 18 active natural orbitals in the DMRG-CASSCF calculations of the $^{1}\{Fe[TIMEN](NO)\}^{-}$ complex (singlet state) with isovalue = 0.04. The number next to each orbital represents the occupation number

Results for $\{Fe[nacnac](NO)_2\}$

Table S62: Electronic energies E (in E_h) and spin populations for Fe, N, O, and NO for $^2\{Fe[nacnac](NO)_2\}$ (doublet state), calculated using different DFT functionals and DMRG-CASSCF

Method	Energy (E_h)	Spin Population			
		Fe	N	O	NO
B3LYP	-2291.006724	2.850	-0.550	-0.481	-1.031
B97-D	-2291.485738	1.784	-0.281	-0.226	-0.507
BP86	-2292.023535	1.327	-0.152	-0.124	-0.276
M06-L	-2291.441482	2.383	-0.415	-0.375	-0.790
M06	-2290.810231	2.986	-0.568	-0.524	-1.092
PBE0	-2290.200928	3.152	-0.621	-0.549	-1.170
PBE	-2290.238677	1.315	-0.148	-0.121	-0.269
TPSSh	-2291.750605	2.335	-0.420	-0.346	-0.765
TPSS	-2291.941835	1.393	-0.170	-0.134	-0.304
DMRG-CASSCF	-2284.257484	1.480	-0.140	-0.110	-0.251

Table S63: Geometric data for $^2\{Fe[nacnac](NO)_2\}$, calculated using BP86

d(Fe-N) (Å)	d(N-O) (Å)	ν(N-O) (cm^{-1})
1.657/1.666	1.179/1.181	1741/1775

Table S64: Comparison between DMRG-CASSCF results with m = 1000 and 1500 in $^2\{Fe[nacnac](NO)_2\}$

	$m = 1000$ (Fiedler ordering)	$m = 1000$ (GA ordering)	$m = 1500$
Energy(E_h)^a	−2284.257484	-	−2284.259206
Energy(E_h)^b	−2284.251607	−2284.253225	−2284.256406
Weights (in percentage) of configurations			
$[\pi^*(NO)]^0$	0.05	0.06	0.06
$[\pi^*(NO)]^1$	1.85	1.87	1.88
$[\pi^*(NO)]^2$	20.41	20.39	20.31
$[\pi^*(NO)]^3$	50.29	50.16	50.04
$[\pi^*(NO)]^4$	22.98	22.90	22.91
$[\pi^*(NO)]^5$	0.73	0.73	0.75
Other	3.68	3.88	4.06

^{a}Energies calculated with CheMPS2, natural orbitals, Fiedler ordering ^{b}Energies calculated with BLOCK2, localized orbitals. The value at $m = 1500$ was calculated with Genetic Algorithm (GA) ordering.

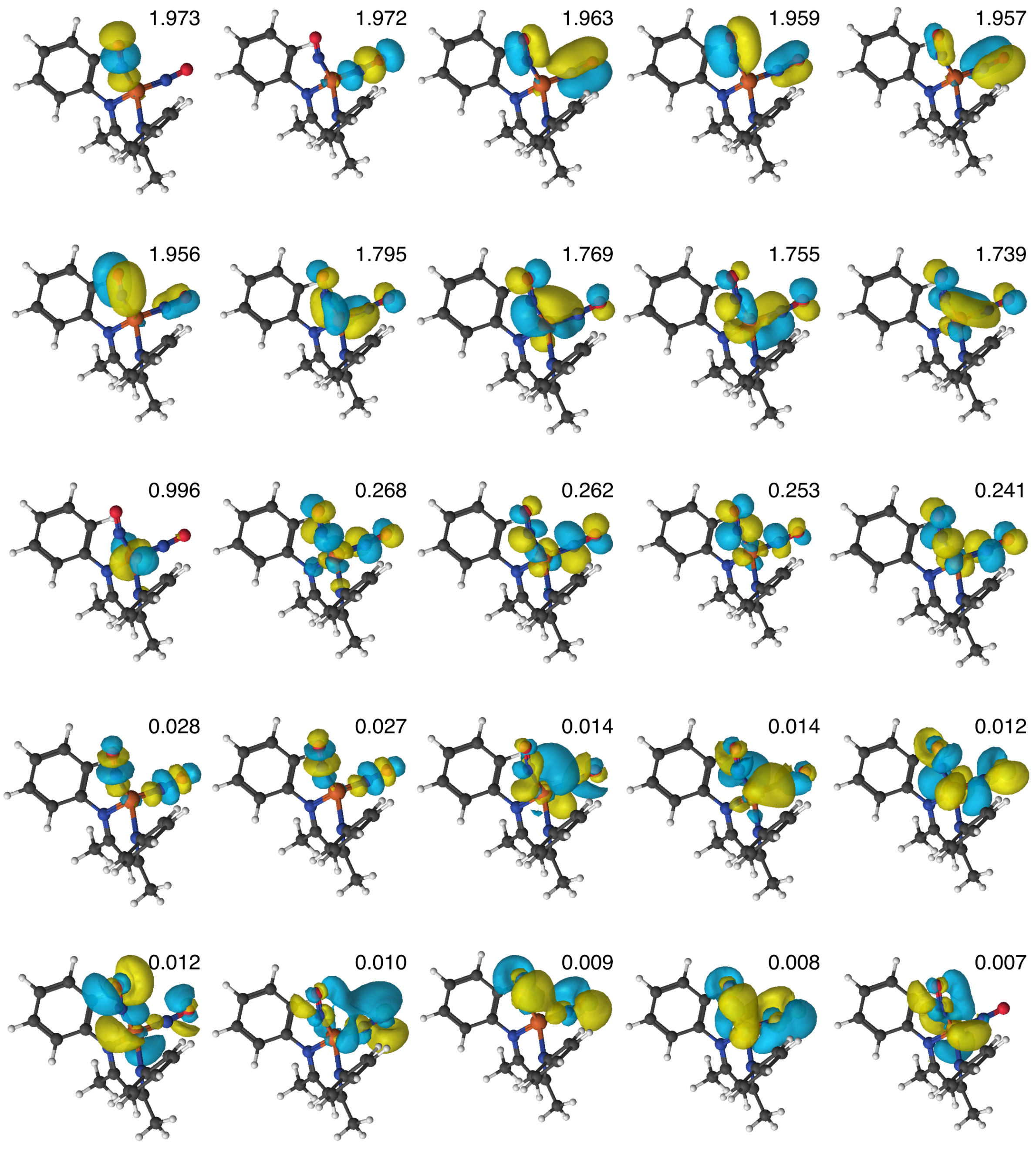

Figure S16: Representations of the 25 active natural orbitals in the DMRG-CASSCF calculations of the $^2\{Fe[nacnac](NO)_2\}$ complex (doublet state) with isovalue = 0.04. The number next to each orbital represents the occupation number

Results for $\{Fe[nacnac](NO)_2\}^-$

Table S65: Electronic energies E (in E_h) and spin populations for Fe, N, O, and NO for $^1\{Fe[nacnac](NO)_2\}^-$ (singlet state), calculated using different DFT functionals and DMRG-CASSCF

Method	Energy (E_h)	Spin Population			
		Fe	N	O	NO
B3LYP	-2291.050655	0	0	0	0
B97-D	-2291.544222	0	0	0	0
BP86	-2292.091593	0	0	0	0
M06-L	-2291.489451	0	0	0	0
M06	-2290.842041	0	0	0	0
PBE0	-2290.230706	0	0	0	0
PBE	-2290.302690	0	0	0	0
TPSSh	-2291.805721	0	0	0	0
TPSS	-2292.003563	0	0	0	0
DMRG-CASSCF	-2284.286786	0	0	0	0

Table S66: Geometric data for $^1\{Fe[nacnac](NO)_2\}^-$, calculated with BP86.

d(Fe-N) (Å)	d(N-O) (Å)	ν(N-O) (cm^{-1})
1.657/1.666	1.179/1.181	1741/1775

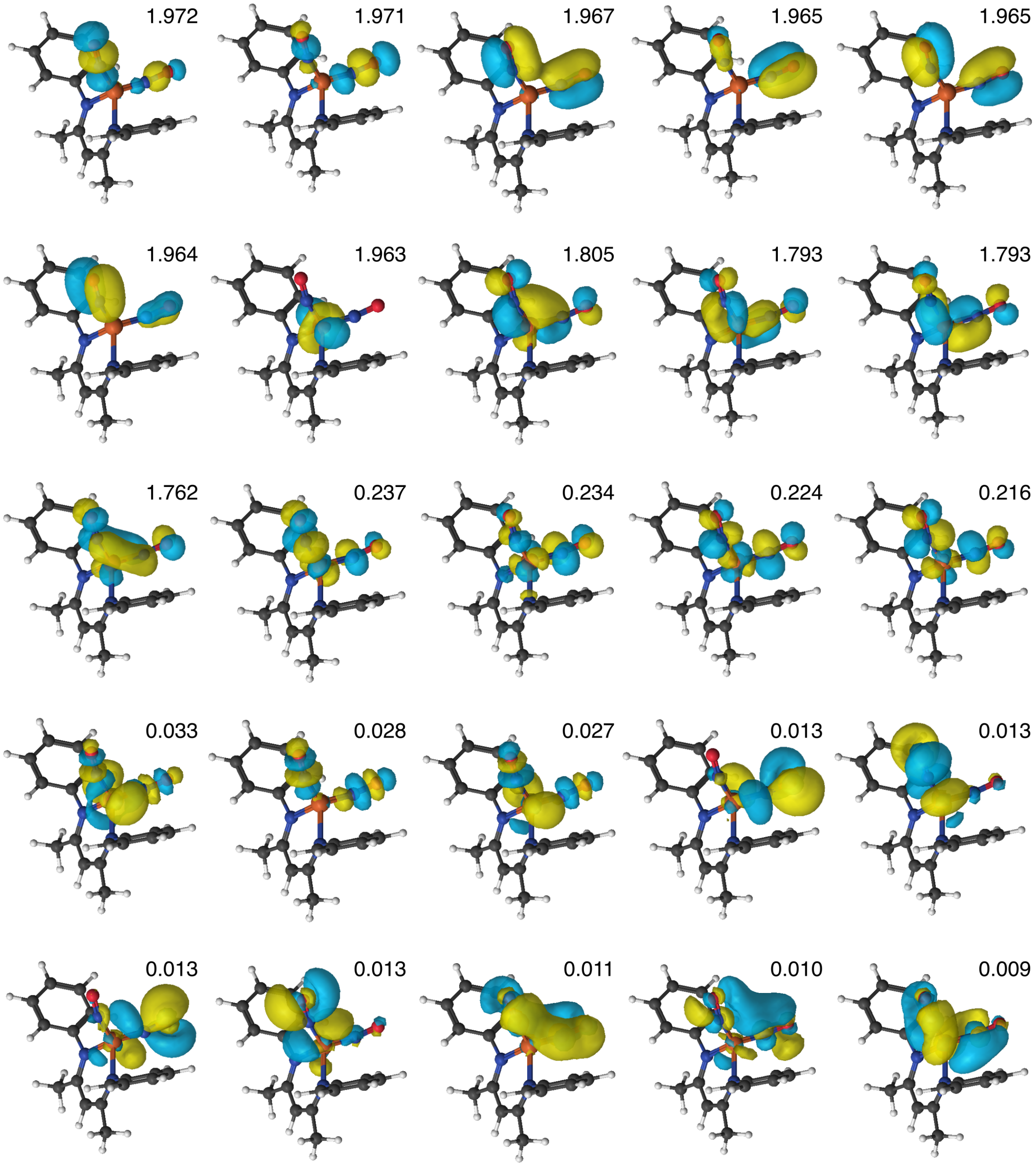

Figure S17: Representations of the 25 active natural orbitals in the DMRG-CASSCF calculations of the $^1\{Fe[nacnac](NO)_2\}^-$ complex (singlet state) with isovalue = 0.04. The number next to each orbital represents the occupation number

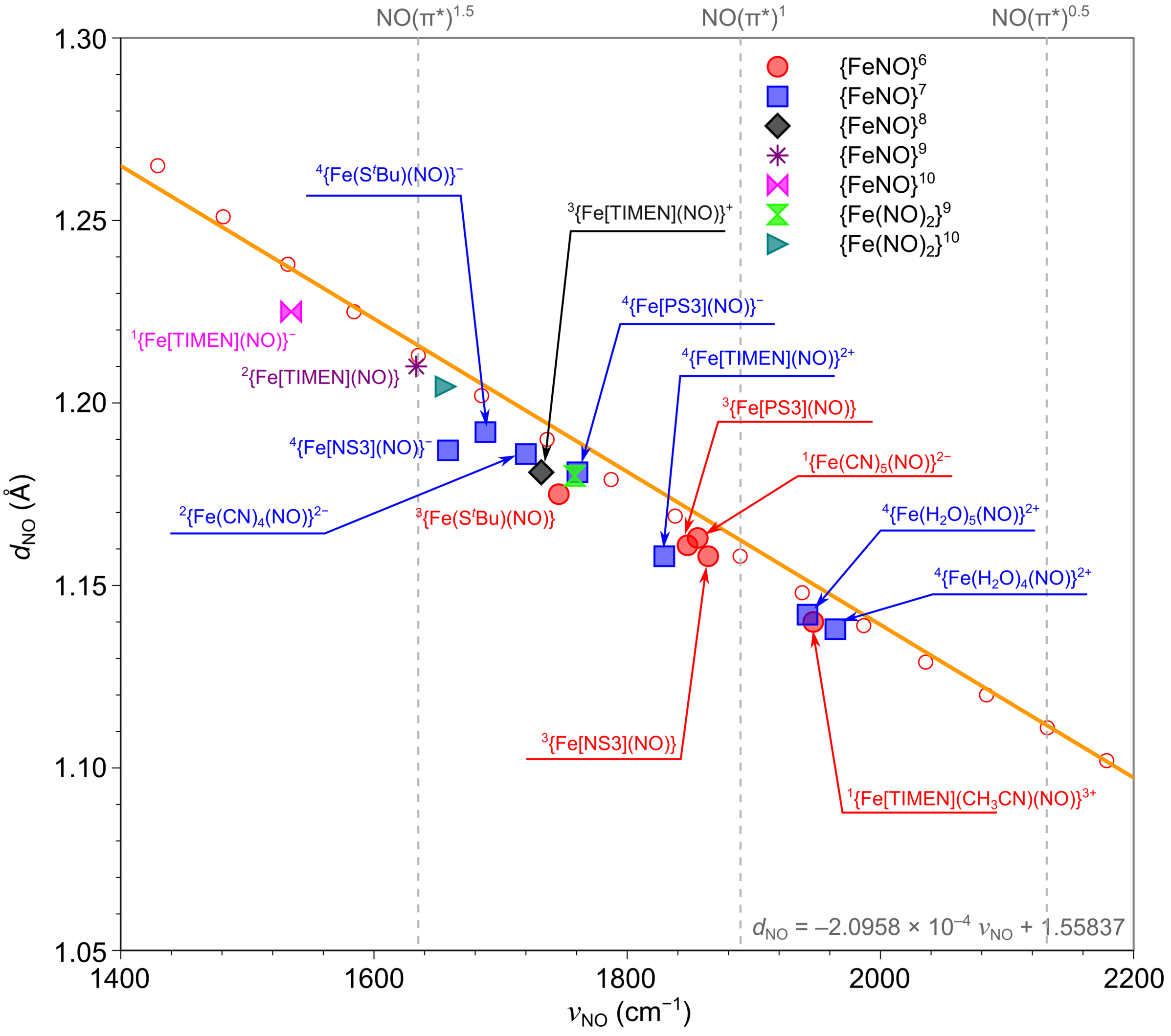

Figure S18: Correlation between NO bond distance and vibrational frequency, calculated at the BP86-D3(BJ)/def2-TZVP level of theory. The orange line is the calibration curve, obtained by a linear fit to the frequencies of isolated NO molecules with different NO(π^*) occupancies, represented by open circles.
